# Differential IL18 signaling via IL18 receptor and Na-Cl co-transporter discriminating thermogenesis and glucose metabolism regulation

**DOI:** 10.1038/s41467-022-35256-8

**Published:** 2022-12-08

**Authors:** Xian Zhang, Songyuan Luo, Minjie Wang, Qiongqiong Cao, Zhixin Zhang, Qin Huang, Jie Li, Zhiyong Deng, Tianxiao Liu, Cong-Lin Liu, Mathilde Meppen, Amelie Vromman, Richard A. Flavell, Gökhan S. Hotamışlıgil, Jian Liu, Peter Libby, Zhangsuo Liu, Guo-Ping Shi

**Affiliations:** 1 School of Food and Biological Engineering, Hefei University of Technology, Hefei, Anhui 230009 China; 2 Department of Medicine, Brigham and Women’s Hospital and Harvard Medical School, Boston, MA 02115 USA; 3 Department of Cardiology, Guangdong Cardiovascular Institute, Guangdong Provincial People’s Hospital, Guangdong Academy of Medical Sciences, Guangzhou, Guangdong 510000 China; 4 Department of Nephrology, the First Affiliated Hospital, Research Institute of Nephrology, Zhengzhou University, Henan Province Research Center For Kidney Disease, Key Laboratory of Precision Diagnosis and Treatment for Chronic Kidney Disease in Henan Province, Zhengzhou, Henan 450052 China; 5 Department of Immunobiology, Yale School of Medicine, New Haven, CT 06520 USA; 6 Department of Molecular Metabolism, Harvard School of Public Health, Boston, MA 02115 USA

**Keywords:** Obesity, Diabetes, Interleukins, Lymphocyte differentiation

## Abstract

White adipose tissue (WAT) plays a role in storing energy, while brown adipose tissue (BAT) is instrumental in the re-distribution of stored energy when dietary sources are unavailable. Interleukin-18 (IL18) is a cytokine playing a role in T-cell polarization, but also for regulating energy homeostasis via the dimeric IL18 receptor (IL18r) and Na-Cl co-transporter (NCC) on adipocytes. Here we show that IL18 signaling in metabolism is regulated at the level of receptor utilization, with preferential role for NCC in brown adipose tissue (BAT) and dominantly via IL18r in WAT. In *Il18r*^−/−^*Ncc*^−/−^ mice, high-fat diet (HFD) causes more prominent body weight gain and insulin resistance than in wild-type mice. The WAT insulin resistance phenotype of the double-knockout mice is recapitulated in HFD-fed *Il18r*^−/−^ mice, whereas decreased thermogenesis in BAT upon HFD is dependent on NCC deletion. BAT-selective depletion of either NCC or IL18 reduces thermogenesis and increases BAT and WAT inflammation. IL18r deletion in WAT reduces insulin signaling and increases WAT inflammation. In summary, our study contributes to the mechanistic understanding of IL18 regulation of energy metabolism and shows clearly discernible roles for its two receptors in brown and white adipose tissues.

## Abstract (web summary)

Interleukin-18 (IL18) has a pivotal role in interferon signalling and T cell development, but increasingly recognized as an adipokine that regulates energy metabolism in fat tissue. Authors here dissect the function of IL18 signalling in the adipose compartment by targeted genomic deletion of its two receptors individually and in combination in brown and white adipose tissues.

## Introduction

In humans and mice, white adipose tissue (WAT) stores excess chemical energy in the form of triglyceride, whereas brown adipose tissue (BAT) dissipates stored chemical energy in the form of heat^1,2^. White adipocyte dysfunction contributes to obesity and type-2 diabetes^3^. Insulin signaling plays a major role in glucose uptake and lipid metabolism in mature white adipocytes^4^. Insulin signaling is essential for adipocyte differentiation and preadipocyte survival^5,6^. Brown and beige adipocyte generation and recruitment in BAT or WAT contribute to energy homeostasis and protects against obesity^7,8^. Immune-adipose crosstalk is essential to the activation of brown/beige adipose tissues. Studies have suggested a role for inflammatory cytokines in regulating adipose tissue thermogenesis. Interleukin-4 (IL4) and IL13 from eosinophils activate thermogenesis in the adipose tissue upon stimulation by cold and exercise^9,10^. IL33 activates type-2 innate lymphoid cells and licenses uncoupled respiration to promote beige fat biogenesis and UCP1 (uncoupling protein-1) splicing by binding to its ST2 receptor^11–13^. Recent studies showed that IL10 acted on IL10Rα to repress thermogenic gene expression in adipocytes^14^.

Earlier studies showed that IL18 polarizes T cells and induces IFN-γ production^15^. IL18 is also found in non-T cells, including adipocytes^16–19^. Several studies highlighted the importance of IL18 in regulating obesity and energy homeostasis. IL18 concentrations are elevated in obese and diabetic subjects^20–23^. Circulating IL18 levels correlate with bodyweight, adiposity, insulin resistance, hypertriglyceridemia, and metabolic syndrome in human and mice^24–27^. Yet, IL18 deficiency in mice led to hyperphagia, obesity, insulin resistance, and decreased energy expenditure^28–31^. IL18-binding protein (IL18BP) is a naturally occurred IL18 inactivator^32^. Similar phenotypes to those of IL18-deficient (*Il18*^−/−^) mice were obtained from IL18BP-overexpressing mice^28^. Exogenous IL18 administration reduced bodyweight regain, appetite, feed efficiency and induced inguinal subcutaneous fat (iWAT) browning^28–30^. Therefore, IL18 is thought a homeostatic regulator that is elevated compensatively in obesity to oppose excess energy, analogous to insulin and adipokine leptin^30^. Yet, this hypothesis and the possible underlying mechanisms remain uncertain. IL18 binds to IL18 receptor (IL18r) that consists of a α-chain responsible for ligand binding and a β-chain for signaling transduction^33^. IL18r-deficient (*Il18r*^−/−^) mice showed “conflicting” responses to dietary obesity and adipose tissue thermogenesis compared with the *Il18*^−/−^ mice^29,34^. Global deletion of IL18r increased bodyweight in mice on a chow diet but not those on a high-fat diet (HFD)^34^. IL18r deficiency increased lean body mass, iWAT browning, alternative macrophage activation in iWAT from mice on a HFD, but decreased energy expenditure from mice on a chow diet^29^. These differential modulatory functions between IL18r- and IL18-deficient mice in the thermogenic program suggest additional mechanisms of IL18 signaling in adipose tissues. We reported that Na-Cl co-transporter (NCC) acts as an alternative IL18 receptor in atherogenesis^35^. Therefore, IL18 signaling appears beyond the IL18-IL18r interaction. NCC action may explain these divergent responses in dietary obesity and adipose tissue thermogenesis between the *Il18*^−/−^ and *Il18r*^−/−^ mice.

Here we report a role for NCC- and IL18r-mediated IL18 activities in BAT thermogenesis and WAT insulin sensitivity that mitigates obesity and diabetes. IL18 uses NCC to promote BAT thermogenesis and mitochondrial gene expression, but uses IL18r in WAT to enhance glucose sensitivity and insulin signaling. We tested these hypotheses using mice with global deficiency of IL18r or NCC, combined deficiency of IL18r and NCC, BAT-selective deficiency of NCC or IL18, and WAT-selective deficiency of IL18r on a HFD or a low-fat diet (LFD). This study establishes the two coordinate axes of IL18 functions in regulating thermogenesis and insulin sensitivity and furnishes the interaction between IL18 signaling and different adipose tissue functions.

## Results

### Adipose tissue expression of IL18, IL18r, and NCC in response to thermogenic activation and different diets

**Fig. 1:**
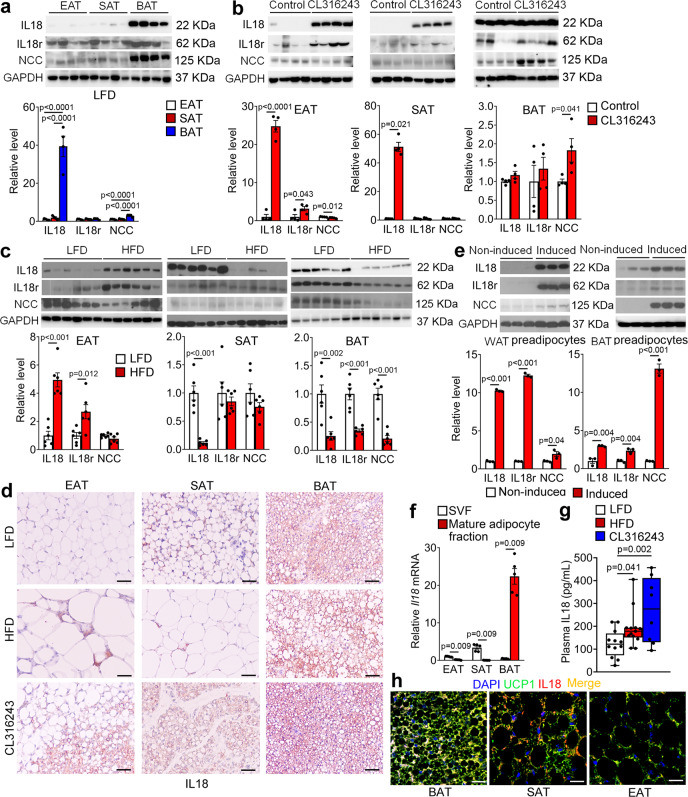
Adipose tissue expression of IL18, IL18r, and NCC in response to thermogenic activation and high-fat diet (HFD. **a–c**. Immunoblot analysis and quantification of IL18, IL18r, and NCC relative to GAPDH in EAT, SAT, and BAT from WT mice on a low fat diet (LFD) (*n* = 4 per group) (**a**), after thermogenic activation with 1 mg/day/kg CL316243 i.p. injection for 7 days (*n* = 4 per group) (**b**), or fed a HFD for 12 weeks (*n* = 6 per group) (**c**). **d** IL18 immunostaining of representative epididymal adipose tissue (EAT), subcutaneous adipose tissue (SAT) and brown adipose tissue (BAT) sections from mice fed a LFD, a HFD, or treated with CL316243 *(n* = 8 per group); scale bar: 50 μm. **e** Immunoblot analysis and quantification of IL18, IL18r, and NCC relative to GAPDH in differentiated white or brown adipocytes (*n* = 3 per group). **f** RT-PCR analysis of IL18 expression in stromal vascular fraction (SVF) or mature adipocyte fractions from EAT, SAT, BAT from WT mice on a LFD (*n* = 5 per group). **g** Plasma IL18 levels from mice fed a LFD, a HFD, or treated with CL316243 (LFD: *n* = 12; HFD: *n* = 13; CL316243: *n* = 8). **h** Immunofluorescent double staining of UCP1 (green) and IL18 (red) in BAT, SAT, or EAT from mice fed a LFD. Representative of 3 independent experiments. Scale: 25 μm. Data are mean ± SEM, two-sided Mann-Whitney U test (**e, f**), two-sided Student’s t-test (**b**, **c**), and one-way ANOVA, followed by LSD post-test (**a**, **g**). Sample sizes were all biologically independent samples.

To determine the source of IL18 and to explore IL18 actions in different adipose tissues, we performed immunoblot analyses and detected the expression of IL18, IL18r, and NCC in different adipose tissues from mice under different treatments. The specificities of antibodies against IL18, IL18r, and NCC have been previously confirmed^35,36^. From mice under a LFD, IL18 and NCC were enriched in BAT, but negligibly in epididymal adipose tissue (EAT) and subcutaneous adipose tissue (SAT). Yet, the IL18r level was comparable in EAT, SAT, and BAT (Fig. 1a). The expression of IL18, IL18r, and NCC in EAT, SAT, and BAT was regulated in response to thermogenic activation and HFD. From mice treated with a β3-adrenoceptor (β3-AR) agonist CL316243 for 7 days, IL18 expression in EAT and SAT was increased, whereas IL18 expression in BAT was consistently high with or without CL316243 stimulation (Fig. 1b). IL18r was increased in EAT and NCC was significantly increased in BAT after CL316243 treatment (Fig. 1b). We detected similar expression regulation of IL18, IL18r, and NCC in adipose tissues from mice after 7 days of cold exposure at 4 °C, which acts on both β1-AR and β3-AR to activate adipose tissue thermogenesis^37^. IL18 expression was increased in EAT and SAT and remained consistently high in BAT with or without cold stimulation. IL18r was also increased in SAT and NCC was increased in SAT and BAT after cold exposure (Supplementary Fig. 1). After mice were fed a HFD for 12 weeks, however, we found that both IL18 and IL18r were increased in EAT, IL18 was reduced in SAT, and IL18, IL18r and NCC were all reduced in BAT (Fig. 1c).

Results from immunostaining yielded the same conclusions. IL18 and NCC were expressed in the multilocular beige or brown adipocytes. Their expression in EAT, SAT, and BAT was decreased when mice were on a HFD, but increased after mice received CL316243 treatment. IL18 and IL18r were found in crown-like cells in response to HFD (Fig. 1d and Supplementary Fig. 2). Differentiation of pre-adipocytes from the stromal vascular fractions (SVFs) of WAT (Fig. 1e, left) or BAT (Fig. 1e, right) increased the expression of IL18, IL18r, and NCC. To determine the cellular source of IL18, we dispersed EAT, SAT, and BAT and separated the mature adipocytes from pre-adipocytes in the SVFs. Use of RT-PCR detected IL18 mostly in the SVFs from EAT and SAT, but in mature adipocytes from BAT (Fig. 1f). We also found that HFD and CL316243 treatment led to a significant increase of plasma IL18 (Fig. 1g). Immunofluorescent staining revealed colocalization of IL18 and UCP1 in BAT, but at lower levels in SAT and negligible level in EAT from mice fed a LFD (Fig. 1h). Together, these observations suggest that the UCP1-positive brown adipocytes in BAT and beige cells in SAT are the major source of IL18. Thermogenic activation and HFD feeding alter the expression profiles and possibly activities of IL18 and its two receptors IL18r and NCC.

### Combined deficiency of IL18r and NCC aggravates HFD-induced obesity and insulin resistance

**Fig. 2:**
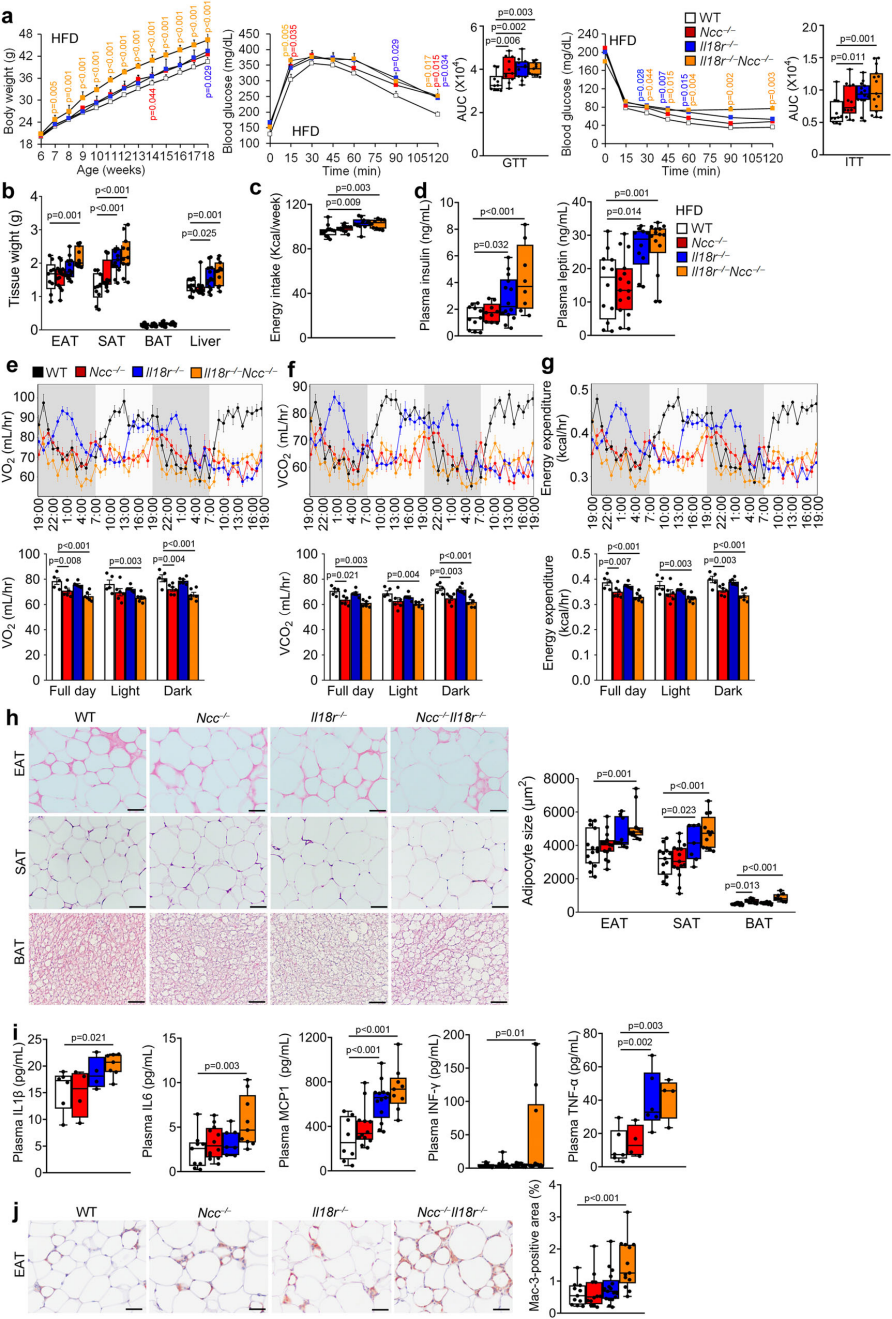
Deficiency of IL18r and NCC aggravates obesity, insulin resistance and inflammation in mice fed a HFD. **a**–**d** Bodyweight gain, glucose tolerance test (GTT), AUC of GTT, insulin tolerance test (ITT), AUC of ITT (WT: *n* = 10; *Ncc*^−/−^: *n* = 11, *Il18r*^−/−^: *n* = 11; *Il18r*^−/−^*Ncc*^−/−^: *n* = 11) (**a**), EAT, SAT, BAT, and liver weights (WT: *n* = 9; *Ncc*^−/−^: *n* = 14, *Il18r*^−/−^: *n* = 13; *Il18r*^−/−^*Ncc*^−/−^: *n* = 11) (**b**), energy intake (WT: *n* = 12; *Ncc*^−/−^: *n* = 11, *Il18r*^−/−^: *n* = 10; *Il18r*^−/−^*Ncc*^−/−^: *n* = 10) (**c**), and plasma insulin (WT: *n* = 11; *Ncc*^−/−^: *n* = 10, *Il18r*^−/−^: *n* = 13; *Il18r*^−/−^*Ncc*^−/−^: *n* = 8) and leptin (WT: *n* = 12; *Ncc*^−/−^: *n* = 15, *Il18r*^−/−^: *n* = 10; *Il18r*^−/−^*Ncc*^−/−^: *n* = 14) levels (**d**) from HFD-fed WT, *Ncc*^−/−^, *Il18r*^−/−^, and *Il18r*^−/−^*Ncc*^−/−^ mice for 12 weeks. **e**–**g** Mouse metabolic parameters, including oxygen consumption (VO_2_) (**e**), carbon dioxide production (VCO_2_) (**f**), and energy expenditure (**g**) and their average values from full day cycle, light cycle, and dark cycle during 48 hrs of monitoring in WT, *Ncc*^−/−^, *Il18r*^−/−^, and *Il18r*^−/−^*Ncc*^−/−^ mice (WT: *n* *=* 5; *Ncc*^−/−^: *n* = 6, *Il18r*^−/−^: *n* = 6;* Il18r*^−/−^*Ncc*^−/−^: *n* = 6). **h** Representative images of hematoxylin and eosin (H&E) staining and adipocyte sizes in EAT, SAT, and BAT from indicated mouse groups (WT: *n* = 14; *Ncc*^−/−^: *n* = 15, *Il18r*^−/−^: *n* = 9; *Il18r*^−/−^*Ncc*^−/−^: *n* = 12); scale bar: 50 μm. **i**. Plasma IL1β (WT: *n* = 6; *Ncc*^*−/−*^: *n* = 4, *Il18r*^*−/−*^: *n* = 4; *Il18r*^*−/−*^*Ncc*^*−/−*^: *n* = 7), IL6 (WT: *n* = 9; *Ncc*^*−/−*^: *n* = 12, *Il18r*^*−/−*^: *n* = 7; *Il18r*^*−/−*^*Ncc*^*−/−*^: *n* = 9), MCP1 (WT: *n* = 8; *Ncc*^*−/−*^: *n* = 12, *Il18r*^*−/−*^: *n* = 13; *Il18r*^*−/−*^*Ncc*^*−/−*^: *n* = 9), IFN-γ (WT: *n* = 10; *Ncc*^*−/−*^: *n* = 16, *Il18r*^*−/−*^: *n* = 12; *Il18r*^*−/−*^*Ncc*^*−/−*^*: n* = 14), and TNF-α (WT: *n* = 6; *Ncc*^*−/−*^: *n* = 4, *Il18r*^*−/−*^: *n* = 6; *Il18r*^*−/−*^*Ncc*^*−/−*^: *n* = 4) levels from indicated mouse groups. **j** Mac-3 immunostaining of representative EAT sections and quantification from indicated mouse groups (WT: *n* = 11; *Ncc*^*−/−*^: *n* = 12, *Il18r*^*−/−*^: *n* = 17; *Il18r*^*−/−*^*Ncc*^*−/−*^: *n* = 12); scale: 50 μm. Data are mean ± SEM, two-way ANOVA repeated-measures (**a**) or one-way ANOVA test (**b-j**), followed by LSD post-test. Sample sizes were all biologically independent samples.

Differential expression regulation of IL18r and NCC in adipose tissues (EAT, SAT, and BAT) in response to thermogenic stimulation and HFD suggests their participation in obesity and insulin resistance. To test the role and dissect the mechanism of IL18 activities in systemic metabolic homeostasis, we fed wild-type (WT), *Il18r*^−/−^, *Ncc*^−/−^, and *Il18r*^−/−^*Ncc*^−/−^ littermate mice a HFD for 12 weeks. Compared with the WT mice, *Il18r*^−/−^ and *Ncc*^−/−^ mice showed significant but moderate differences in bodyweight gain, glucose tolerance test (GTT) and the AUC (area under the curve) of GTT, and insulin tolerance test (ITT) and the AUC of ITT. In contrast, the *Il18r*^−/−^*Ncc*^−/−^ mice demonstrated the highest bodyweight gain and the worst glucose intolerance and insulin resistance (Fig. 2a). *Il18r*^−/−^*Ncc*^−/−^ mice also showed the highest increases of EAT, SAT, and liver tissue weight, whereas *Il18r*^−/−^ mice only showed higher SAT and liver weight than those of WT mice (Fig. 2b). Single deficiency of IL18r or combined deficiency of IL18r and NCC, but not a single deficiency of NCC in mice increased energy intake (Fig. 2c) and plasma insulin and leptin levels (Fig. 2d). To assess the IL18 activity in thermogenesis, we assessed the energy expenditure in HFD-fed WT, *Ncc*^−/−^, *Il18r*^−/−^, and *Il18r*^−/−^*Ncc*^−/−^ mice. Each mouse was individually housed in a metabolic chamber for 48 hrs. The consumption rate (VO_2_), carbon dioxide production (VCO_2_), and energy expenditure of these mice were measured. The results showed that compared with the WT control mice, *Ncc*^−/−^ and *Il18r*^−/−^*Ncc*^−/−^ mice showed reduced full day and dark cycle VO_2_ (Fig. 2e), VCO_2_ (Fig. 2f), and energy expenditure (Fig. 2g) after 12 weeks of HFD. Only *Il18r*^−/−^*Ncc*^−/−^ mice but not *Ncc*^−/−^ and *Il18r*^−/−^ mice showed significant reductions of light cycle VO_2_, VCO_2_ and energy expenditure (Fig. 2e–g). Histological analyses revealed bigger adipocytes in EAT, SAT, and BAT and many fewer multilocular lipid droplet-containing cells in BAT from *Il18r*^−/−^*Ncc*^−/−^ mice than in WT mice. Adipocyte sizes were also enlarged in SAT from *Il18r*^−/−^ mice and in BAT from *Ncc*^−/−^ mice (Fig. 2h). Combined deficiency of IL18r and NCC markedly increased plasma pro-inflammatory cytokine and chemokine levels, including IL1β, IL6, MCP1, IFN-γ, and TNF-α (Fig. 2i), and Mac-3-positive macrophage numbers in EAT (Fig. 2j). *Il18r*^−/−^ mice also showed higher plasma MCP1 and TNF-α levels than WT mice. NCC deficiency did not affect any of these plasma inflammatory molecules (Fig. 2i). These findings suggest a cooperative action of IL18r and NCC in mediating the protective function of IL18 in HFD-induced obesity and insulin resistance. NCC in BAT may mediate the IL18 function in brown adipocytes and energy expenditure, whereas IL18r in WAT may be responsible for the IL18 activity in systemic inflammation.

### Divergent roles of NCC and IL18r in mediating IL18 activities in BAT and WAT

**Fig. 3:**
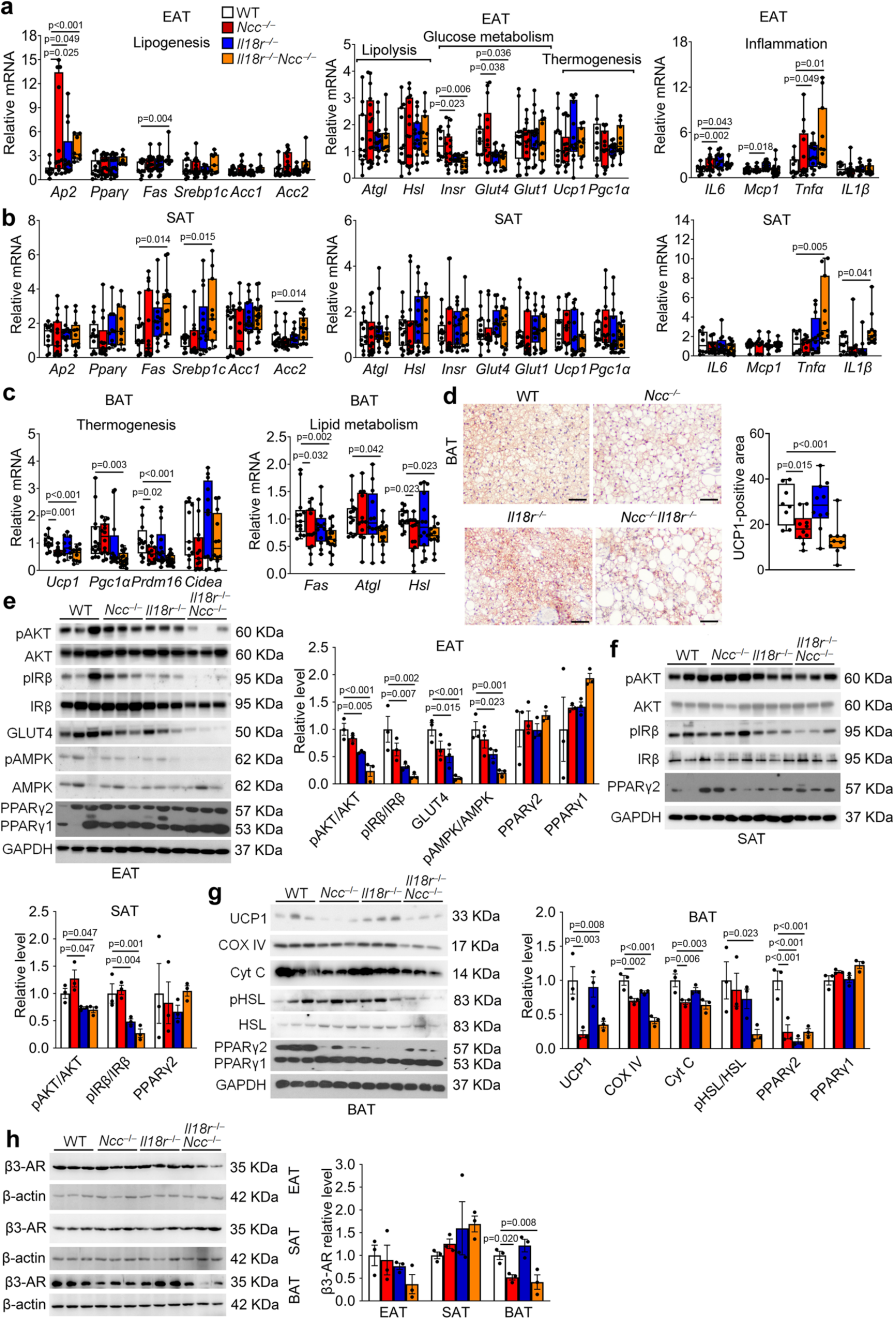
Deficiency of IL18r and NCC interrupts WAT lipid metabolism, glucose metabolism, insulin signaling, and inflammation, and impairs BAT lipolysis and thermogenesis from mice fed a HFD. **a**–**c** RT-PCR analysis of lipogenic, lipolytic, glucose metabolic, thermogenic, or inflammatory genes in EAT (WT: *n* = 10; *Ncc*^*−/−*^: *n* = 16, *Il18r*^*−/−*^: *n* = 16; *Il18r*^*−/−*^*Ncc*^*−/−*^: *n* = 9) (**a**), SAT (WT: *n* = 12; *Ncc*^*−/−*^: *n* = 15, *Il18r*^*−/−*^: *n* = 13; *Il18r*^*−/−*^*Ncc*^*−/−*^: *n* = 11) (**b**), and BAT (WT: *n* = 13; *Ncc*^*−/−*^: *n* = 13, *Il18r*^*−/−*^: *n* = 11; *Il18r*^*−/−*^*Ncc*^*−/−*^: *n* = 12) (**c**) in WT, *Ncc*^*−/−*^, *Il18r*^*−/−*^, and *Il18r*^*−/−*^*Ncc*^*−/−*^ mice after 12 weeks of a HFD. **d**. UCP1 immunostaining of representative BAT sections and quantification in the indicated groups of mice (WT: *n* = 8; *Ncc*^*−/−*^: *n* = 10, *Il18r*^*−/−*^: *n* = 10; *Il18r*^*−/−*^*Ncc*^*−/−*^: *n* = 10); scale: 50 μm. **e**–**h** Immunoblot analysis and quantification of pAKT, pIRβ, GLUT4, pAMPK, and PPARγ relative to total AKT, IRβ, AMPK, or GAPDH in EAT (**e**), pAKT, pIRβ, and PPARγ relative to total AKT, IRβ, or GAPDH in SAT (**f**), and UCP1, COX IV, Cyt C, pHSL, and PPARγ, relative to total HSL or GAPDH in BAT (**g**), and β3-AR relative to β-actin in EAT, SAT, and BAT (**h**) from indicated mouse groups (*n* = 3). Data are mean ± SEM, one-way ANOVA test, followed by LSD post-test. Sample sizes were all biologically independent samples.

To evaluate the specific functions of IL18r and NCC in IL18 signaling, we tested whether NCC and IL18r single or combined deficiency affected adipocyte biology in different adipose tissues. We first assessed the expression of genes that are associated with lipogenesis, lipolysis, glucose metabolism, thermogenesis, and inflammation. In EAT, NCC deficiency or combined deficiency upregulated the mRNA levels of lipid metabolic genes *Ap2* and *Fas*. IL18r deficiency or combined deficiency downregulated the expression of glucose metabolic genes *Insr* and *Glut4*. In IL18r- or NCC-deficient or IL18r/NCC combined deficient mice, the expressions of inflammatory molecules *IL6*, *Mcp1*, and *Tnfα* were increased. Yet, the expression of IL18r or NCC did not affect the expression of thermogenic genes *Ucp1* and *Pgc1α* and lipolysis genes *Atgl* and *Hsl* (Fig. 3a). In SAT, the expressions of lipid metabolic genes *Fas*, *Srebp1c*, and *Acc2* were increased in *Il18r*^*−/−*^*Ncc*^*−/−*^ mice, but not in *Il18r*^*−/−*^ or *Ncc*^*−/−*^ mice. The expression of thermogenic, lipolytic, and glucose metabolic genes in SAT did not differ among the four groups of mice. The expression of inflammatory cytokine genes *Tnfα* and *IL1β* were increased in *Il18r*^*−/−*^*Ncc*^*−/−*^ mice, but not in *Il18r*^*−/−*^ or *Ncc*^*−/−*^ mice (Fig. 3b). In BAT, NCC deficiency or combined deficiency of both receptors downregulated the expression of thermogenic genes *Ucp1*, *Pgc1α*, and *Prdm16* and lipid metabolic genes *Fas*, *Atgl*, and *Hsl* (Fig. 3c). UCP1 immunostaining revealed significantly decreased UCP1-positive areas in BAT from *NCC*^*−/−*^ and *Il18r*^*−/−*^*Ncc*^*−/−*^ mice (Fig. 3d). These observations suggest that NCC deficiency impairs lipolysis and thermogenesis in BAT but IL18r deficiency may interfere glucose metabolism and insulin signaling in EAT from HFD-induced obese mice. To test these possibilities, we examined the expression and activation of insulin signaling molecules protein kinase B (AKT) and insulin receptor β (IRβ), glucose metabolism molecules glucose transporter type-4 (GLUT4) and AMP-activated protein kinase (AMPK) in different adipose tissues from different mice. Immunoblot analyses showed that AKT, IRβ, and AMPK activation and GLUT4 expression were all significantly decreased in EAT from *Il18r*^*−/−*^ and *Il18r*^*−/−*^*Ncc*^*−/−*^ mice, but not in those from *Ncc*^*−/−*^ mice (Fig. 3e). AKT and IRβ activations were similarly reduced in SAT from *Il18r*^*−/−*^ and *Il18r*^*−/−*^*Ncc*^*−/−*^ mice, but not in SAT from *Ncc*^*−/−*^ mice (Fig. 3f). The expression of IL18r or NCC did not affect the expression of PPARγ in EAT or SAT (Fig. 3e,f). In contrast, the expressions of UCP1 and mitochondrial proteins cytochrome C oxidase-IV (COX IV) and cytochrome C (Cyt C) were decreased in BAT from *Ncc*^*−/−*^ and *Il18r*^*−/−*^*Ncc*^*−/−*^ mice, but not in those from *Il18r*^*−/−*^ mice (Fig. 3g). The activation of hormone-sensitive lipase (HSL) was downregulated in BAT from *Il18r*^*−/−*^*Ncc*^*−/−*^ mice, but not in those from single receptor-deficient mice (Fig. 3g). Interestingly, single deficiency of IL18r or NCC, or combined deficiency of IL18r and NCC all blocked the expression of PPARγ2 but not PPARγ1 in BAT (Fig. 3g). Moreover, we tested the expression of β3-AR in different adipose tissues from HFD-fed WT, *Il18r*^*−/−*^, *Ncc*^*−/−*^, and *Il18r*^*−/−*^*Ncc*^*−/−*^ mice. Immunoblot analysis showed that β3-AR expression was similar in EAT and SAT among WT, *Il18r*^*−/−*^, *Ncc*^*−/−*^, and *Il18r*^*−/−*^*Ncc*^*−/−*^ mice. In BAT, however, deficiency of NCC or both receptors reduced β3-AR expression (Fig. 3h). These observations support the hypothesis that NCC mediates IL18 functions in energy expenditure by regulating mitochondrial functions and thermogenesis in BAT, whereas IL18r mediates IL18 activities in glucose tolerance and insulin sensitivity by regulating insulin signaling and glucose metabolism in EAT.

### NCC but not IL18r mediates IL18 activities in thermogenesis, mitochondrial activity, and lipolysis in BAT

**Fig. 4:**
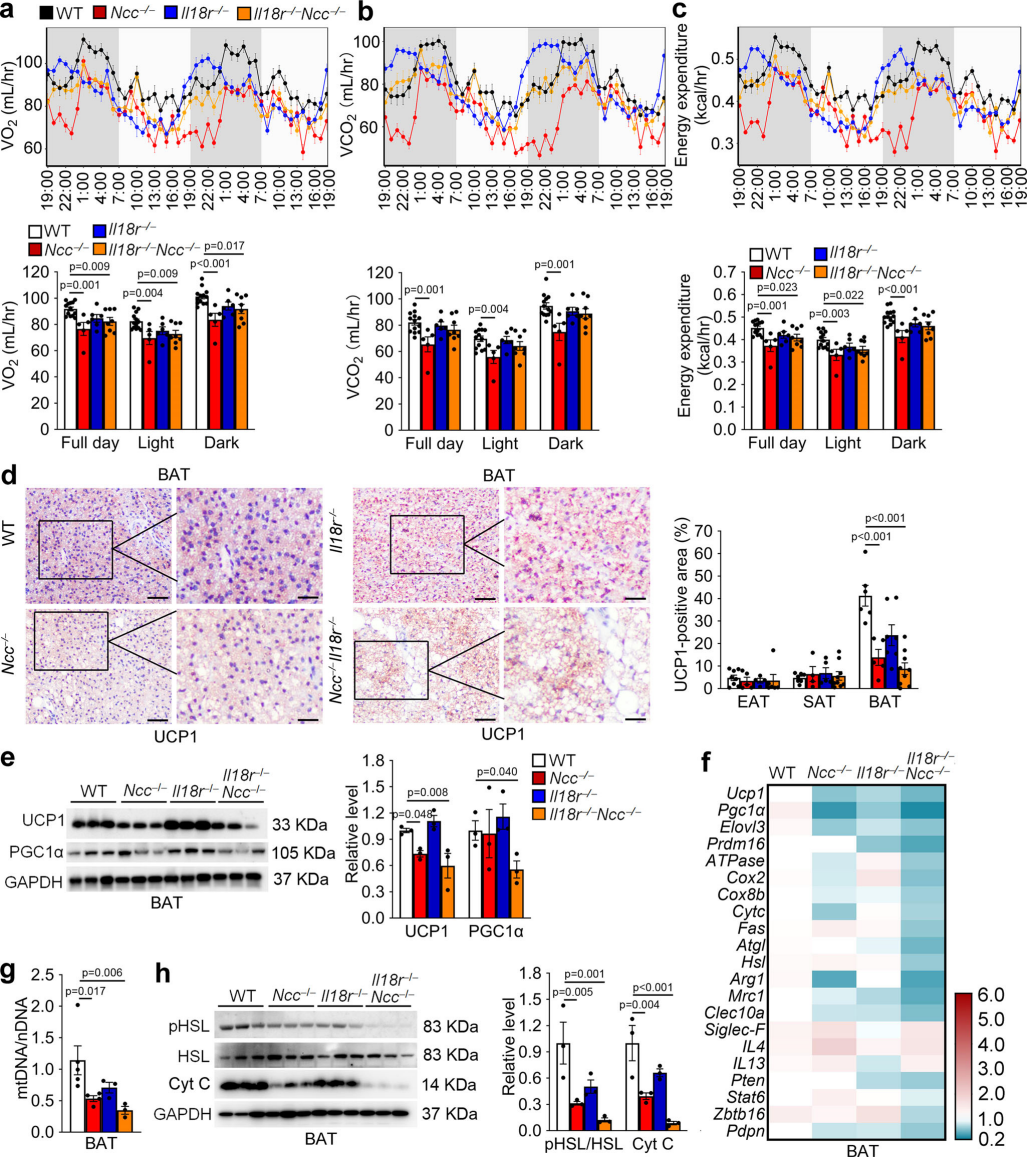
Impaired energy expenditure, and decreased lipolysis and mitochondrial activity in BAT from *Ncc*^*−/−*^ and *Il18r*^*−/−*^*Ncc*^*−/−*^ mice in response to CL316243. **a**–**c** Mouse metabolic parameters, including oxygen consumption (VO_2_) (**a**), carbon dioxide production (VCO_2_) (**b**), and energy expenditure (**c**) and their average values from full day cycle, light cycle, and dark cycle during 48 hrs of monitoring in WT, *Ncc*^*−/−*^, *Il18r*^*−/−*^, and *Il18r*^*−/−*^*Ncc*^*−/−*^ mice after 7 days of CL316243 treatment (WT: *n* = 13; *Ncc*^*−/−*^: *n* = 5, *Il18r*^*−/−*^: *n* = 6; *Il18r*^*−/−*^*Ncc*^*−/−*^: *n* = 8). **d**. Representative images of UCP1 immunostaining of BAT sections and UCP1-positive area in EAT, SAT, and BAT from indicated groups of mice (WT: *n* = 7; *Ncc*^*−/−*^: *n* = 5, *Il18r*^*−/−*^: *n* = 5; *Il18r*^*−/−*^*Ncc*^*−/−*^: *n* = 6). Scale: 50 μm, inset: 25 µm. **e** Immunoblot analysis and quantification of UCP1 and PGC1α relative to GAPDH in BAT from indicated groups of mice (*n* = 3). **f** RT-PCR analysis of thermogenic, mitochondrial genes, lipogenic genes, lipolytic genes, M2 macrophage markers, eosinophil markers, and type 2 cytokines in BAT from indicated groups of mice (WT: *n* = 8; *Ncc*^*−/−*^: *n* = 5, *Il18r*^*−/−*^: *n* = 6; *Il18r*^*−/−*^*Ncc*^*−/−*^: *n* = 8). **g** RT-PCR analysis of BAT mitochondrial DNA (mtDNA) contents (mtDNA/nuclear (n)DNA ratio) in indicated groups of mice (WT: *n* = 5; *Ncc*^*−/−*^: *n* = 4, *Il18r*^*−/−*^: *n* = 3; *Il18r*^*−/−*^*Ncc*^*−/−*^: *n* = 3). **h** Immunoblot analysis and quantification of pHSL and Cyt C relative to total HSL or GAPDH in BAT from indicated groups of mice (*n* = 3). Data are mean ± SEM, one-way ANOVA test, followed by LSD post-test. Sample sizes were all biologically independent samples.

To confirm the role for NCC in IL18 activity in thermogenesis, we first assessed the energy expenditure in LFD-fed WT, *Ncc*^*−/−*^, *Il18r*^*−/−*^, and *Il18r*^*−/−*^*Ncc*^*−/−*^ mice with or without β3-AR agonist CL316243 stimulation for 7 days^38^. Each mouse was individually housed in a metabolic chamber for 48 hrs. Compared with those of WT control mice, *Ncc*^*−/−*^ and *Il18r*^*−/−*^*Ncc*^*−/−*^ mice showed reduced full-day and light cycle oxygen consumption rate (VO_2_) (Fig. 4a). Only *Ncc*^*−/−*^ mice but not *Il18r*^*−/−*^ and *Il18r*^*−/−*^*Ncc*^*−/−*^ mice showed significant reductions of full day, light cycle, and dark cycle carbon dioxide production (VCO_2_) (Fig. 4b). *Ncc*^*−/−*^ and *Il18r*^*−/−*^*Ncc*^*−/−*^ mice showed significant reductions of full day, light cycle, and dark energy expenditure after CL316243 stimulation (Fig. 4c). Yet, VO_2_, VCO_2_, and energy expenditure did not differ among the four groups of mice without CL316243 stimulation (Supplementary Fig. 3a–c). The lean mass and fat mass did not differ between different genotypes of CL316243-treated mice (Supplementary Fig. 4a). Similar to the energy intake of HFD-fed mice, deficiency of IL18r or combined deficiency of IL18r and NCC, but not the single deficiency of NCC increased energy intake in CL316243-treated mice (Supplementary Fig. 4b). Interestingly, the ambulatory activity was lower in *Ncc*^*−/−*^ mice and higher in *Il18r*^*−/−*^ mice than WT mice after CL316243 stimulation (Supplementary Fig. 4c). Morphological analyses and UCP1 immunostaining revealed significantly enlarged multilocular lipid droplets and reduced UCP1-positive areas in BAT from *Ncc*^*−/−*^ and *Il18r*^*−/−*^*Ncc*^*−/−*^ mice, compared with those from WT control mice. Such changes did not occur in EAT and SAT. In contrast, *Il18r*^*−/−*^ mice had comparable adipocyte size and UCP1-positive area to those of WT mice in BAT, EAT, and SAT (Fig. 4d and Supplementary Fig. 4d,e).

Immunoblot analysis yielded the same conclusion. The expression of thermogenic gene UCP1 in BAT from *Ncc*^*−/−*^ and *Il18r*^*−/−*^*Ncc*^*−/−*^ mice, but not that from *Il18r*^*−/−*^ mice reduced significantly, compared with that from WT control mice, although the expression of the mitochondrial thermogenic gene PGC1α was decreased only in BAT from *Il18r*^*−/−*^*Ncc*^*−/−*^ mice (Fig. 4e). Consistent with these results, BAT from *Ncc*^*−/−*^ and *Il18r*^*−/−*^*Ncc*^*−/−*^ mice exhibited decreased mRNA levels of thermogenic genes *Ucp1*, *Pgc1α*, *Elovl3*, and *Prdm16* (Fig. 4f). Such differences did not occur in SAT or EAT (Supplementary Fig. 4f–g). Results from RT-PCR showed that very few thermogenic genes (*Pgc1α*, *Prdm16*), beige adipocyte progenitor gene (*Pdgfrα*), beige adipocyte markers (*CD137*, *Cited1*) (Supplementary Fig. 4f) in EAT, and beige adipocyte marker (*Cited1*) (Supplementary Fig. 4g) in SAT were decreased in *Ncc*^*−/−*^ or/and *Il18r*^*−/−*^*Ncc*^*−/−*^ mice.

Expansion of mitochondrial respiration in brown adipocytes after thermogenic activation was accompanied by an increase of lipid turnover, lipolysis, and de novo lipogenesis^39,40^. After CL316243 activation, the expression of mitochondrial genes *ATPase*, *Cox2*, *Cox8b*, and *Cytc* (Fig. 4f) and mitochondrial DNA contents (Fig. 4g) decreased in BAT from *Ncc*^*−/−*^ and *Il18r*^*−/−*^*Ncc*^*−/−*^ mice, but not in those from *Il18r*^*−/−*^ mice. There were no significant changes in mitochondrial gene expressions in EAT (Supplementary Fig. 4f) and SAT (Supplementary Fig. 4g) from these mice. Immunoblot analyses showed that the activation of HSL, a critical lipolytic enzyme, and expression of mitochondrial Cyt C were significantly reduced in BAT from *Ncc*^*−/−*^ and *Il18r*^*−/−*^*Ncc*^*−/−*^ mice, compared with those from WT control mice. Such reductions in BAT from *Il18r*^*−/−*^ mice did not reach statistical significance (Fig. 4h). RT-PCR showed that the mRNA levels of lipolysis genes *Atgl*, *Hsl* and lipogenesis gene *Fas* were decreased only in BAT from *Il18r*^*−/−*^*Ncc*^*−/−*^ mice (Fig. 4f).

M2 macrophage recruitment^41^, type-2 cytokine signaling^9^, Stat6/Pten axis in regulatory T cells (Treg)^42^, and Zbtb16 expression in PLZF-positive γδ T cells^43^ all have been implicated in the activation of BAT thermogenesis and WAT browning. RT-PCR detected whether the decreases in thermogenesis in *Ncc*^*−/−*^ and *Il18r*^*−/−*^*Ncc*^*−/−*^ mice might affect the responses of these immune cells in different adipose tissues. BAT from *Ncc*^*−/−*^ or *Il18r*^*−/−*^*Ncc*^*−/−*^ mice, but not *Il18r*^*−/−*^ mice showed lower levels of M2 markers *Arg1*, *Mrc1*, *Clec10a* than those of WT control mice. Yet the expressions of eosinophil marker *Siglec-F*, and type-2 cytokines *IL4* and *IL13* did not differ among the groups (Fig. 4f). *Il18r*^*−/−*^*Ncc*^*−/−*^ mice also showed decreased eosinophil marker *Siglec-F*, and type-2 cytokines *IL4* and *IL13* in EAT (Supplementary Fig. 4f) and M2 marker *Clec10a* in SAT (Supplementary Fig. 4g). The expressions of Treg and γδ T-cell functional molecules *Pten*, *Stat6*, *Zbtb16* did not differ significantly in EAT and SAT (Supplementary Fig. 4f,g) among these different types of mice. These observations suggest that NCC played a dominant role in IL18 regulation of thermogenesis in BAT. NCC deficiency impairs thermogenic activity and decreases lipolysis, mitochondrial activity, and M2 macrophage recruitment in BAT.

### IL18 uses NCC in brown adipocytes for thermogenesis and IL18r in white adipocytes for insulin signaling

**Fig. 5:**
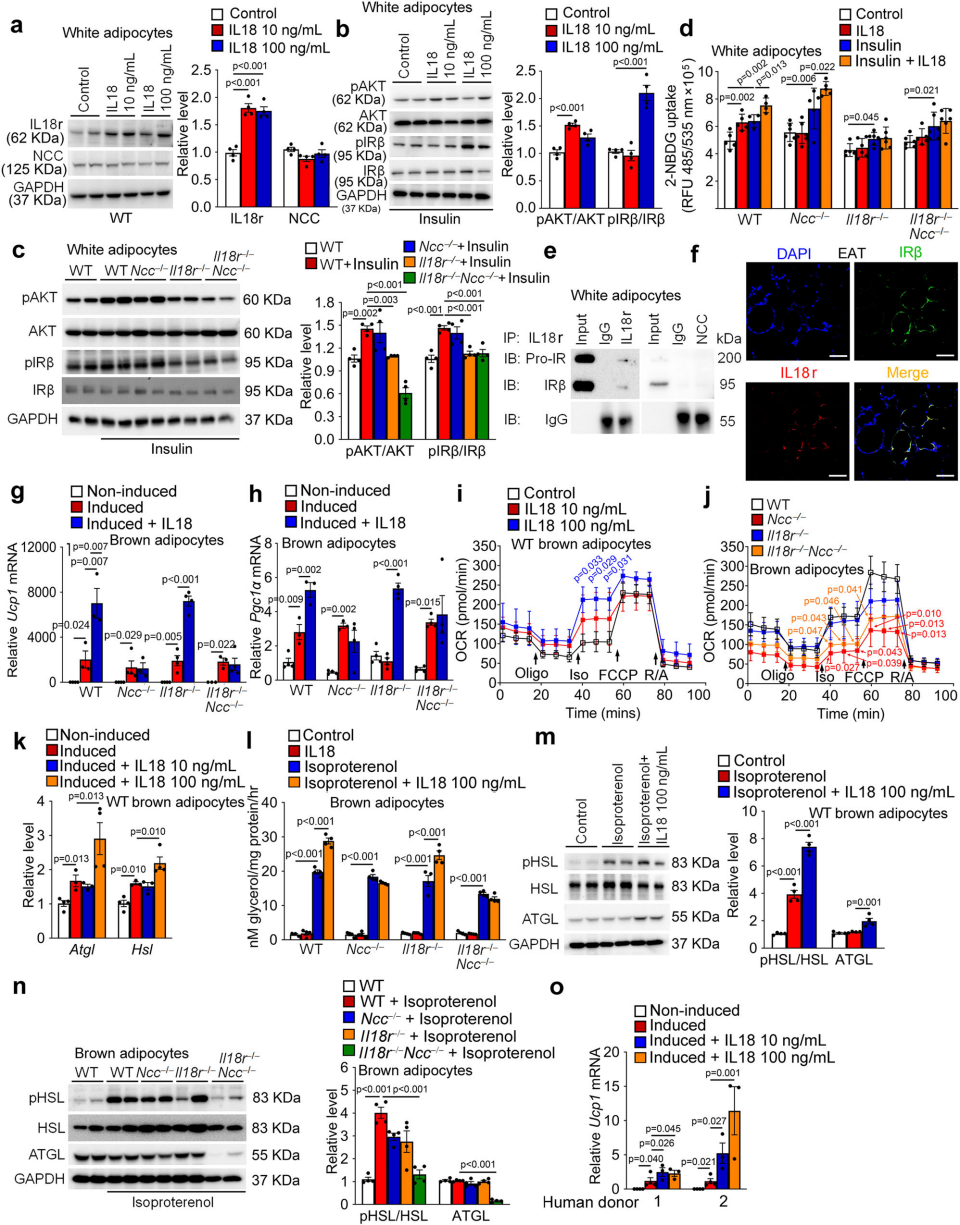
IL18 mediates thermogenesis through NCC in brown adipocytes and insulin signaling through IL18r in white adipocytes. **a** Immunoblots and quantification of IL18r and NCC relative to GAPDH in differentiated WT white adipocytes with or without 10 or 100 ng/mL IL18 for 24 hrs. **b-c** Immunoblots and quantification of pAKT and pIRβ relative to total AKT or IRβ in differentiated white adipocytes from WT mice (**b**) or different types of mice (**c**) treated with or without insulin and IL18 for 30 min. **d** 2-NBDG uptake in differentiated white adipocytes with or without 20 nM insulin and 100 ng/mL of IL18 for 24 hrs. **e** Immunoprecipitation of differentiated white adipocytes lysates (250 µg) with anti-IL18r and anti-NCC antibodies, followed by immunoblot detection of IRβ relative to IgG isotype. **f** Immunofluorescent double staining of IRβ (green) and IL18r (red) in EAT from LFD-fed WT mice. Scale: 25 μm. Representative of 3 independent experiments (**e**–**f**). **g**–**h** RT-PCR analysis of *Ucp1* (**g**) and *Pgc1α* (**h**) in non-differentiated or differentiated brown adipocytes from different mice and treated with or without 100 ng/mL of IL18 for 24 hrs (*n* = 4/group biologically independent samples). **i-j**. Kinetic OCR (oxygen consumption rate) of differentiated white adipocytes from WT mice (**i**) or different types of mice (**j**) in response to oligomycin, isoproterenol, FCCP, and rotenone with antimycin A. **k**. RT-PCR analysis of lipolytic genes in non-differentiated or differentiated brown adipocytes treated with or without IL18 for 24 hrs. **l-n**. Conditional media glycerol level (**l**), immunoblot and quantification of pHSL and ATGL relative to total HSL or GAPDH in WT brown adipocytes (**m**), and pHSL and ATGL relative to total HSL or GAPDH in brown adipocytes from different mice (**n**) treated with or without isoproterenol or IL18 for 3 hrs. **o**. RT-PCR analysis of *Ucp1* levels in human non-differentiated or differentiated brown adipocytes treated with or without IL18 for 24 hrs. *n* = 4/group biologically independent samples. Data are mean ± SEM, one-way ANOVA test, followed by LSD post-test.

To study the mechanisms by which IL18 regulates thermogenesis in brown adipocytes and insulin sensitivity in white adipocytes, we added recombinant IL18 to cultured mouse brown and white adipocytes. Pre-adipocytes in SVFs from WAT (Supplementary Fig. 5a) and BAT (Supplementary Fig. 5b) from WT, *Il18r*^*−/−*^, *Ncc*^*−/−*^, and *Il18r*^*−/−*^*Ncc*^*−/−*^ mice did not differ in adipogenesis, as assessed by oil-red O staining. In cultured white adipocytes, 10 and 100 ng/mL IL18 induced IL18r expression by 2-fold, but displayed no activity on NCC expression (Fig. 5a). IL18 enhanced the activation of insulin-induced AKT and IRβ in these white adipocytes (Fig. 5b). White adipocytes from *Il18r*^*−/−*^ and *Il18r*^*−/−*^*Ncc*^*−/−*^ mice, but not those from *Ncc*^*−/−*^ mice, showed decreased activations of insulin-induced AKT and IRβ, compared with cells from WT mice (Fig. 5c). IL18 also induced glucose (2-NBDG: 2-[N-(7-nitrobenz-2-oxa-1,3-diazol-4-yl) amino]-2-deoxy-D-glucose) uptake with or without insulin activation in WT white adipocytes. Such activity of IL18 was compromised in white adipocytes from *Il18r*^*−/−*^ and *Il18r*^*−/−*^*Ncc*^*−/−*^ mice, but not *Ncc*^*−/−*^ mice (Fig. 5d). However, IL18 did not affect glucose uptake in brown adipocytes from WT, *Il18r*^*−/−*^, *Ncc*^*−/−*^, or *Il18r*^*−/−*^*Ncc*^*−/−*^ mice with or without insulin (Supplementary Fig. 5c). These observations suggest that IL18r, but not NCC dominates the IL18 activity in regulating insulin signaling and glucose uptake in white adipocytes. Immunoprecipitation of white adipocyte lysate from WT mice with anti-IL18r and anti-NCC antibodies followed by IRβ immunoblot analysis demonstrated that pro-IR and IRβ preferentially formed immunocomplexes with the IL18r, but not NCC (Fig. 5e). Immunofluorescent double staining confirmed the colocalization of IL18r and IRβ in epididymal adipocytes (Fig. 5f). Therefore, IL18 may activate insulin signaling by forming the IL18r-IRβ immunocomplexes in white adipocytes.

β-Adrenergic stimulation of brown adipocytes triggers thermogenesis by activating the cAMP-dependent protein kinase (cAMP/PKA) pathway^44^. To examine whether IL18 synergizes this cAMP signaling pathway as a mechanism to stimulate the thermogenesis in brown adipocytes, we assessed the cAMP expression and PKA activation in IL18-treated brown adipocytes. IL18 did not affect these variables in brown adipocytes from WT, *Il18r*^*−/−*^, *Ncc*^*−/−*^, and *Il18r*^*−/−*^*Ncc*^*−/−*^ mice. Only the PKA activity inhibitor H89 decreased the cAMP level in cells from WT mice (Supplementary Fig. 5d,e). Therefore, IL18 may not use the cAMP pathway to stimulate thermogenesis in brown adipocytes. In brown adipocytes, IL18 significantly increased the expression of *Ucp1* and *Pgc1α* after browning differentiation. Single deficiency of NCC, or combined deficiency of IL18r and NCC, but not the single deficiency of IL18r blocked this IL18 activity (Fig. 5g,h). Consistent with these results, cellular respiration seahorse assay demonstrated that IL18 elevated the extracellular oxygen consumption rate (OCR) in WT brown adipocyte, especially the β-adrenergic activator isoproterenol-stimulated uncoupled respiration (Fig. 5i). Brown adipocytes from *Ncc*^*−/−*^ and *Il18r*^*−/−*^*Ncc*^*−/−*^ mice, but not those from *Il18r*^*−/−*^ mice, showed decreased levels of IL18-induced uncoupled respiration (Fig. 5j). IL18 at 100 ng/mL increased the expression of lipolysis genes *Atgl* and *Hsl* in brown adipocytes by RT-PCR (Fig. 5k). IL18 also elevated glycerol release by ELISA (Fig. 5l) and HSL phosphorylation and ATGL expression (Fig. 5m) by immunoblot analysis in brown adipocytes after isoproterenol treatment. Single deficiency of NCC and combined deficiency of IL18r and NCC, but not the single deficiency of IL18r blunted the isoproterenol-induced lipolytic activity (Fig. 5l), HSL phosphorylation, and ATGL expression (Fig. 5n). Human brown adipocytes acted the same as mouse brown adipocytes. Human pre-adipocytes from two donors were induced to brown adipocytes. Recombinant human IL18 at 10 or 100 ng/mL also elevated *Ucp1* expression in differentiated human brown adipocytes (Fig. 5o). These observations suggest that NCC mediates the IL18 activity in thermogenesis, but both IL18r and NCC are required for IL18-controled lipolysis in brown adipocytes. This conclusion is consistent with the observations in BAT from HFD-fed (Fig. 3c–g) and CL316243-treated mice (Fig. 4f–h).

### BAT-selective depletion of IL18 or NCC impairs thermogenesis

**Fig. 6:**
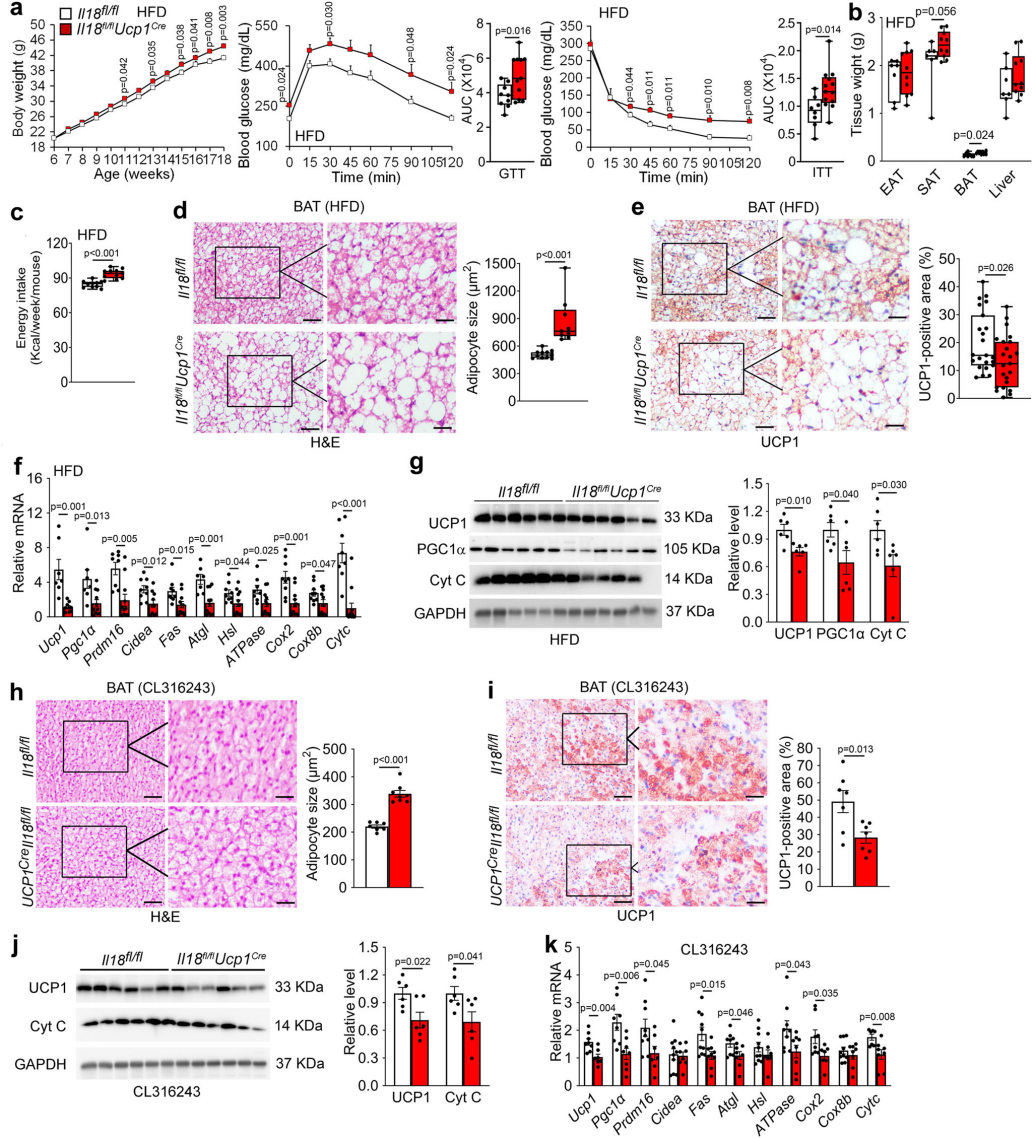
BAT-selective IL18 depletion in *Il18*^*fl/fl*^*Ucp1*^*Cre*^ mice exacerbates obesity and insulin resistance and impairs BAT thermogenesis. **a**–**c** Bodyweight gain (*Il18*^*fl/fl*^: *n* = 10; *Il18*^*fl/fl*^*Ucp1*^*Cre*^: *n* = 13), GTT and AUC of GTT (*Il18*^*fl/fl*^*: n* = 9; *Il18*^*fl/fl*^*Ucp1*^*Cre*^: *n* = 12), ITT and AUC of ITT (*Il18*^*fl/fl*^: *n* = 8; *Il18*^*fl/fl*^*Ucp1*^*Cre*^: *n* = 13) (**a**), EAT, SAT, BAT, and liver weights (*Il18*^*fl/fl*^: *n* = 7; *Il18*^*fl/fl*^*Ucp1*^*Cre*^: *n* = 10) (**b**), and energy intake (*Il18*^*fl/fl*^: *n* = 10; *Il18*^*fl/fl*^*Ucp1*^*Cre*^: *n* = 11) (**c**) in *Il18*^*fl/fl*^ and *Il18*^*fl/fl*^*Ucp1*^*Cre*^ mice fed a HFD for 12 weeks. **d**–**e** H&E (*Il18*^*fl/fl*^: *n* = 11; *Il18*^*fl/fl*^*Ucp1*^*Cre*^: *n* = 9) (**d**) and UCP1 immunostaining (*Il18*^*fl/fl*^: *n* = 23; *Il18*^*fl/fl*^*Ucp1*^*Cre*^: *n* = 23) (**e**) of BAT from indicated groups of mice. Scale: 50 μm. Inset: 25 μm. **f** RT-PCR analysis of thermogenic, lipolytic, and mitochondrial genes expression in BAT from indicated mice (*Il18*^*fl/fl*^: *n* = 8; *Il18*^*fl/fl*^*Ucp1*^*Cre*^: *n* = 11). **g** Immunoblots and quantification of UCP1, PGC1α, and Cyt C relative to GAPDH in BAT from indicated mice (*n* = 6 per group). **h-i**. H&E (**h**) and UCP1 immunostaining (**i**) of BAT from indicated mice (*n* = 7 per group). Scale: 50 μm. Inset: 25 μm. **j.** Immunoblots and quantification of UCP1 and Cyt C relative to GAPDH in BAT from indicated mice (*n* = 6 per group). **k** RT-PCR analysis of thermogenic, lipolytic, and mitochondrial genes in BAT from indicated mice (*n* = 8 per group). Data are mean ± SEM, two-way ANOVA repeated-measures, followed by LSD post-test (**a**), two-sided Student’s t-test (**b**-**c**, **e**-**k**), and two-sided Mann-Whitney *U* test (**d**). Sample sizes were all biologically independent samples.

IL18 and NCC are primarily expressed in BAT in mice on a LFD (Fig. 1a). To test a role for IL18 and NCC in BAT in controlling obesity and glucose intolerance in response to HFD and thermogenic stimulation, we selectively depleted IL18 or NCC in UCP1-expressing brown and beige adipocytes by crossbreeding the *Ucp1*^*Cre*^ mice with the *Il18*^*fl/fl*^ or *Ncc*^*fl/fl*^ mice to generate *Il18*^*fl/fl*^*Ucp1*^*Cre*^ and *Ncc*^*fl/fl*^*Ucp1*^*Cre*^ mice. Immunoblot and RT-PCR analyses affirmed the blunted IL18 expression in BAT from *Il18*^*fl/fl*^*Ucp1*^*Cre*^ mice compared with that in *Il18*^*fl/fl*^ mice. IL18 expression in other tissues such as SAT, liver, and kidney was nearly undetectable (Supplementary Fig. 6a, b). To assess the role of BAT-derived IL18 in HFD-induced obesity and insulin resistance, we fed *Il18*^*fl/fl*^*Ucp1*^*Cre*^ and *Il18*^*fl/fl*^ mice a HFD for 12 weeks. *Il18*^*fl/fl*^*Ucp1*^*Cre*^ mice gained more bodyweight, and showed worse glucose tolerance and insulin resistance than the *Il18*^*fl/fl*^ control mice (Fig. 6a). Tissue analyses revealed that HFD-fed *Il18*^*fl/fl*^*Ucp1*^*Cre*^ mice had higher BAT mass than that of the *Il18*^*fl/fl*^ mice (Fig. 6b). HFD-fed *Il18*^*fl/fl*^*Ucp1*^*Cre*^ mice also showed increased weekly HFD energy intake (Fig. 6c). H&E staining and UCP1 immunostaining characterized BAT lipid droplet sizes and thermogenesis in *Il18*^*fl/fl*^*Ucp1*^*Cre*^ mice. BAT from *Il18*^*fl/fl*^*Ucp1*^*Cre*^ mice showed larger lipid droplets (Fig. 6d) and smaller UCP1-positive areas (Fig. 6e) than those in BAT from the *Il18*^*fl/fl*^ mice. Such differences in lipid droplets did not appear in EAT and SAT from *Il18*^*fl/fl*^*Ucp1*^*Cre*^ and *Il18*^*fl/fl*^ mice (Supplementary Fig. 6c). RT-PCR and immunoblot analyses revealed decreased expression of thermogenic markers *Ucp1*, *Pgc1α*, *Prdm16*, and *Cidea*, lipogenic and lipolytic markers *Fas*, *Atgl*, and *Hsl*, and mitochondrial genes *ATPase*, *Cox2*, *Cox8b*, and *Cytc* in BAT from *Il18*^*fl/fl*^*Ucp1*^*Cre*^ mice (Fig. 6f, g). BAT-selective depletion of IL18 also affected the responses of *Il18*^*fl/fl*^*Ucp1*^*Cre*^ mice to thermogenic activation from β3-AR agonist. Although CL316243 treatment for 7 days did not affect bodyweight or tissue weight (Supplementary Fig. 6d, e) between the *Il18*^*fl/fl*^*Ucp1*^*Cre*^ and *Il18*^*fl/fl*^ mice, H&E staining and UCP1 immunostaining revealed significantly larger lipid droplets and smaller UCP1-positive area in BAT from *Il18*^*fl/fl*^*Ucp1*^*Cre*^ mice than those from *Il18*^*fl/fl*^ mice (Fig. 6h,i). H&E staining did not reveal lipid droplet size differences in EAT and SAT from CL316243-treated *Il18*^*fl/fl*^*Ucp1*^*Cre*^ and *Il18*^*fl/fl*^ mice (Supplementary Fig. 6f), but immunoblot and RT-PCR analyses revealed decreased expression of thermogenic, lipogenic, lipolytic, and mitochondrial genes, including *Ucp1*, *Pgc1α*, *Prdm16*, *Fas*, *Atgl*, *ATPase*, and *Cytc* in BAT from *Il18*^*fl/fl*^*Ucp1*^*Cre*^ mice (Fig. 6j,k). These observations indicate that IL18 in BAT maintains the thermogenic homeostasis and protects mice from obesity and insulin resistance.

**Fig. 7:**
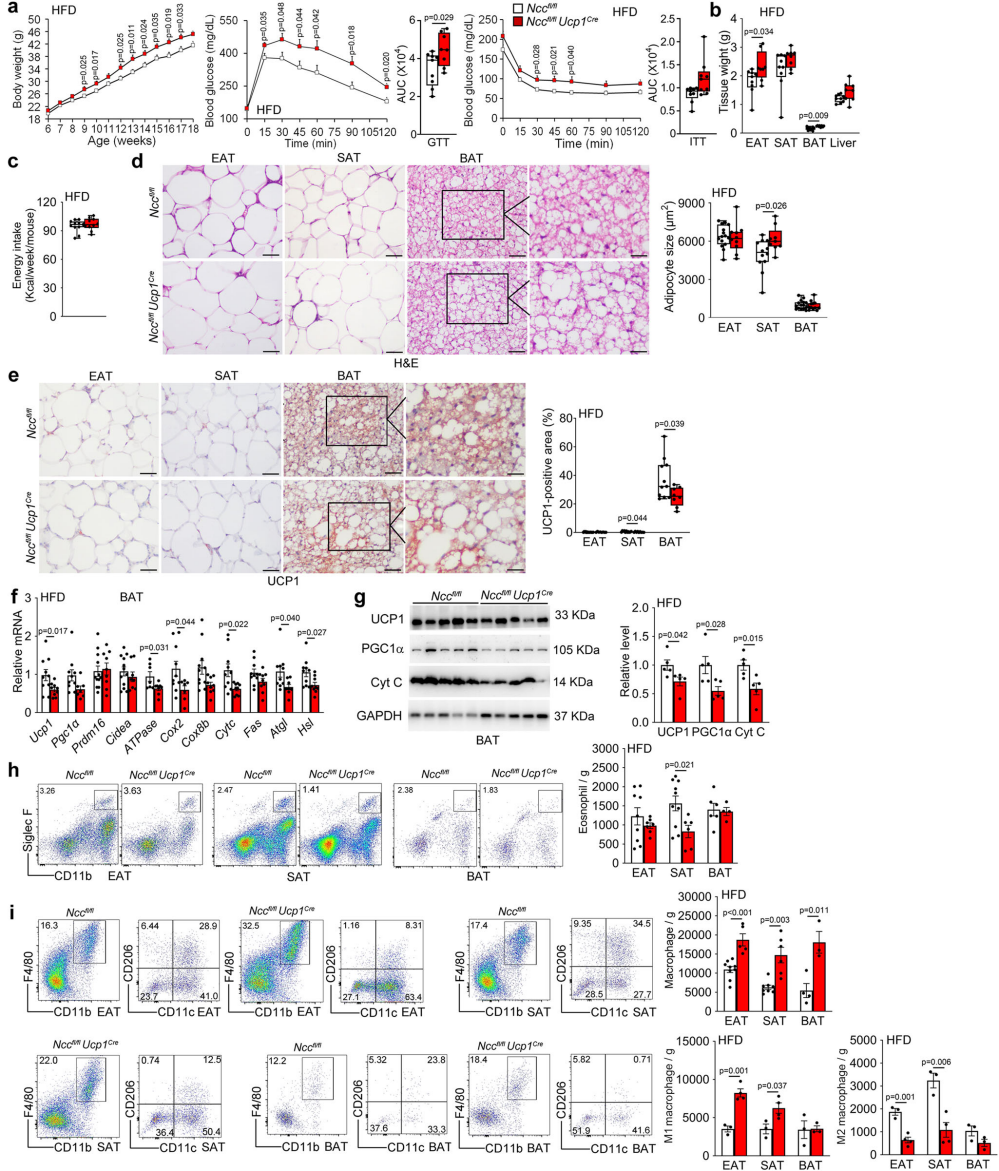
BAT-selective NCC depletion in *Ncc*^*fl/fl*^*Ucp1*^*Cre*^ mice exacerbates obesity and insulin resistance and impairs thermogenesis in HFD-fed mice. **a**–**c** Bodyweight gain (*Ncc*^*fl/fl*^: *n* = 13; *Ncc*^*fl/fl*^*Ucp1*^*Cre*^: *n* = 9), GTT and AUC of GTT (*Ncc*^*fl/fl*^: *n* = 9; *Ncc*^*fl/fl*^*Ucp1*^*Cre*^: *n* = 8), ITT and AUC of ITT (*Ncc*^*fl/fl*^: *n* = 11; *Ncc*^*fl/fl*^*Ucp1*^*Cre*^: *n* = 9) (**a**), EAT, SAT, BAT, and liver weights (*n* = 9 per group) (**b**), and energy intake (*Ncc*^*fl/fl*^: *n* = 11; *Ncc*^*fl/fl*^*Ucp1*^*Cre*^: *n* *=* 10) (**c**) in *Ncc*^*fl/fl*^ and *Ncc*^*fl/fl*^*Ucp1*^*Cre*^ mice fed a HFD for 12 weeks. **d**–**e** H&E (*Ncc*^*fl/fl*^: *n* = 15; *Ncc*^*fl/fl*^*Ucp1*^*Cre*^: *n* = 9) (**d**) and UCP1 immunostaining (*Ncc*^*fl/fl*^: *n* = 11; *Ncc*^*fl/fl*^*Ucp1*^*Cre*^: *n* = 9) (**e**) of EAT, SAT, and BAT from indicated groups of mice. Scale: 50 μm. Inset: 25 μm. **f** RT-PCR analysis of thermogenic, lipolytic, and mitochondrial genes expression in BAT from indicated mice (*Ncc*^*fl/fl*^: *n* = 10; *Ncc*^*fl/fl*^*Ucp1*^*Cre*^: *n* = 9). **g** Immunoblots and quantification of UCP1, PGC1α, and Cyt C relative to GAPDH in BAT from indicated mice (*n* = 5 per group). **h-i**. Representative FACS images and quantification of CD11b^+^Siglec F^+^ eosinophils (*Ncc*^*fl/fl*^-EAT: *n* = 9; *Ncc*^*fl/fl*^*Ucp1*^*Cre*^-EAT: *n* = 7; *Ncc*^*fl/fl*^-SAT: *n* = 10; *Ncc*^*fl/fl*^*Ucp1*^*Cre*^-SAT: *n* = 6; *Ncc*^*fl/fl*^-BAT: *n* = 6; *Ncc*^*fl/fl*^*Ucp1*^*Cre*^-BAT: *n* = 4) (**h**) and CD11b^+^F4/80 ^+^ total, CD11b^+^F4/80 ^+^CD11c^+^ M1, and CD11b^+^F4/80 ^+^CD206^+^ M2 macrophages in EAT, SAT, and BAT (*Ncc*^*fl/fl*^-EAT: *n* = 3; *Ncc*^*fl/fl*^*Ucp1*^*Cre*^-EAT: *n* = 4; *Ncc*^*fl/fl*^-SAT: *n* = 3; *Ncc*^*fl/fl*^*Ucp1*^*Cre*^-SAT: *n* = 4; *Ncc*^*fl/fl*^-BAT: *n* = 3; *Ncc*^*fl/fl*^*Ucp1*^*Cre*^-BAT: *n* = 3) (**i**) from indicated mice. Data are mean ± SEM, two-way ANOVA repeated-measures, followed by LSD post-test (**a**), two-sided Mann-Whitney *U* test (**b, e, f**), two-sided Student’s t-test (**c**, **d**, **g**–**i**). Sample sizes were all biologically independent samples.

Depletion of *Ncc* expression in UCP1-positive brown and beige adipocytes from *Ncc*^*fl/fl*^*Ucp1*^*Cre*^ mice under a HFD was confirmed by RT-PCR. Compared with that in *Ncc*^*fl/fl*^ control mice, *Ncc* expression was blunted in BAT from *Ncc*^*fl/fl*^*Ucp1*^*Cre*^ mice. Low level of *Ncc* expression in SAT, likely from beige cells from *Ncc*^*fl/fl*^*Ucp1*^*Cre*^ mice was also blocked (Supplementary Fig. 7a). After 12 weeks on a HFD, *Ncc*^*fl/fl*^*Ucp1*^*Cre*^mice gained more bodyweight, showed worse glucose intolerance and insulin resistance (Fig. 7a) and had higher EAT and BAT masses (Fig. 7b) than *Ncc*^*fl/fl*^ control mice, but these two groups of mice had comparable weekly energy intake (Fig. 7c). H&E staining showed that SAT but not EAT or BAT from *Ncc*^*fl/fl*^*Ucp1*^*Cre*^ mice had larger lipid droplets than those in *Ncc*^*fl/fl*^ mice (Fig. 7d). UCP1 immunostaining showed that SAT and BAT from *Ncc*^*fl/fl*^*Ucp1*^*Cre*^ mice had smaller UCP1-positive areas (Fig. 7e) than *Ncc*^*fl/fl*^ control mice, consistent to the blunted *Ncc* expression in BAT and SAT from *Ncc*^*fl/fl*^*Ucp1*^*Cre*^ mice (Supplementary Fig. 7a). The expression of thermogenic markers *Ucp1* and *Pgc1α*, lipolytic markers *Atgl*, and *Hsl*, and mitochondrial genes *ATPase*, *Cox2*, and *Cytc* was reduced in BAT from *Ncc*^*fl/fl*^*Ucp1*^*Cre*^ mice by RT-PCR and immunoblot analyses (Fig. 7f–g). BAT-selective depletion of NCC also increased the expression of lipogenic gene *Ap2* and inflammatory cytokines *IL6*, *Mcp1*, and *IL1β* in EAT and decreased the expression of lipolytic markers *Atgl*, and *Hsl* in EAT and *Ucp1* in SAT (Supplementary Fig. 7b, c). Yet, most of the lipogenesis and glucose metabolism genes in EAT (Supplementary Fig. 7b) and SAT (Supplementary Fig. 7c), and AKT and IRβ activation in WAT (Supplementary Fig. 7d) were not affected by NCC depletion in BAT. FACS analyses revealed significantly fewer eosinophils in SAT (Fig. 7h), and M2 macrophages in EAT and SAT, but more total macrophages in EAT, SAT, and BAT, and M1 macrophages in EAT and SAT from *Ncc*^*fl/fl*^*Ucp1*^*Cre*^ mice than those in *Ncc*^*fl/fl*^ control mice after 12 weeks of a HFD (Fig. 7i). Yet, NCC depletion in BAT showed moderate impact on T cells. Only Th2 cells decreased in SAT and Th17 cells increased in EAT and SAT from *Ncc*^*fl/fl*^*Ucp1*^*Cre*^ mice. Total CD8^+^ T cells, CD4^+^ T cells, and Tregs were not affected (Supplementary Fig. 8a–e). IL18 signaling is known to activate eosinophils, macrophages, and T cells^15,35,45^. To assess whether reduced eosinophils in SAT and changes of macrophages in EAT, SAT, and BAT from *Ncc*^*fl/fl*^*Ucp1*^*Cre*^ mice were due to NCC depletion in BAT or secondary to the increased body weight gain, glucose intolerance, and insulin resistance in these mice, we measured the eosinophil, total macrophage, M1 and M2 macrophage, total CD4^+^ and CD8^+^ T-cell, Th1, Th2, Th17, and Treg cell counts in EAT, SAT, and splenocytes from *Ncc*^*fl/fl*^*Ucp1*^*Cre*^ and *Ncc*^*fl/fl*^ mice under a LFD. The results showed that NCC depletion in BAT did not affect any of these cells in SAT, EAT, and spleens from these mice (Supplementary Fig. 9a–e), although we did not perform the same analysis in BAT due to their small tissue size and low inflammatory cell frequency. The gating strategies to detect adipose tissue and splenic macrophages, eosinophils, CD4^+^ and CD8^+^ T cells, Th1, Th2, Th17 T cells, and Treg are shown in Supplementary Fig. 10a–e.

**Fig. 8:**
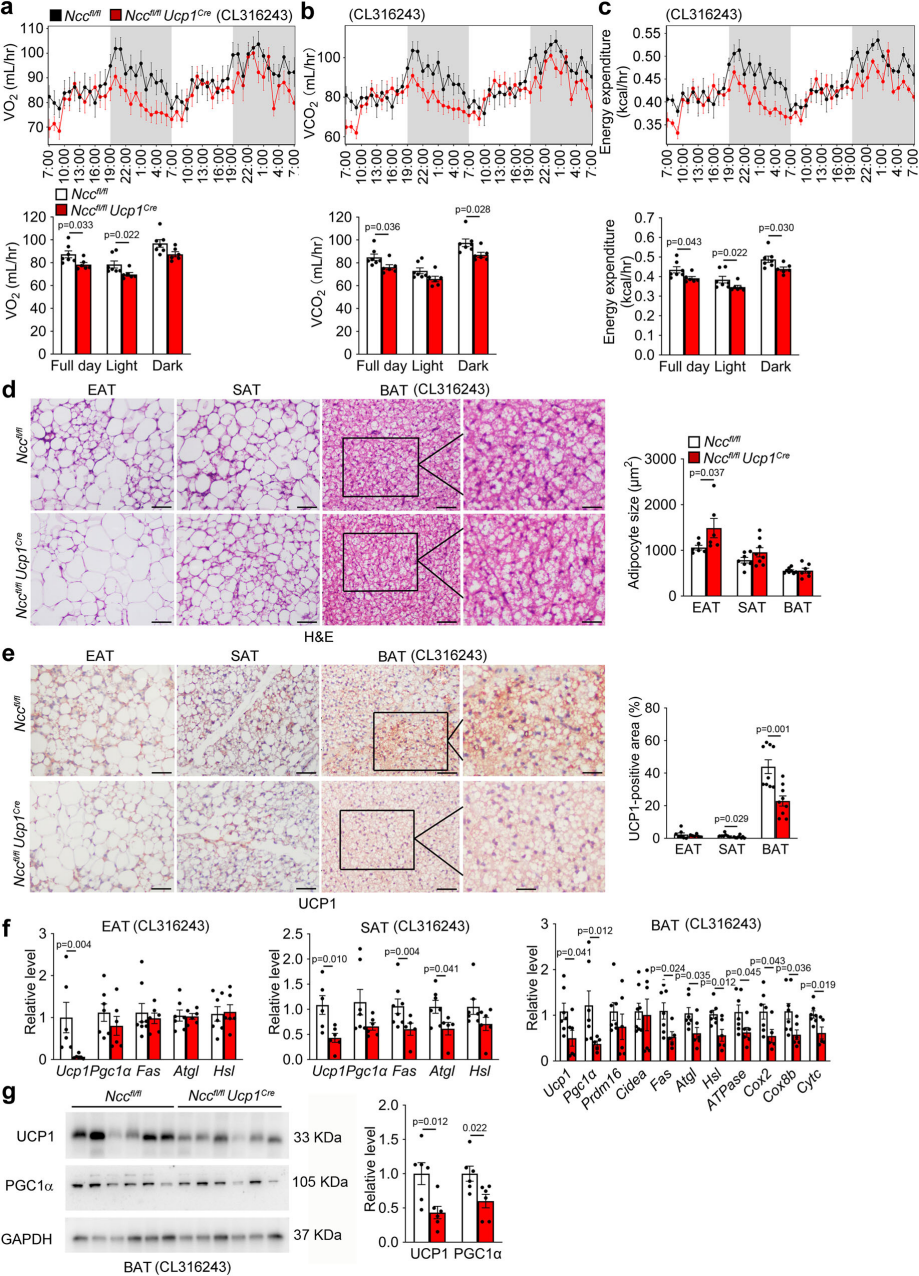
BAT-selective NCC depletion in *Ncc*^*fl/fl*^*Ucp1*^*Cre*^ mice impairs thermogenesis in response to CL316243. **a**–**c** Mouse metabolic parameters, including VO_2_ (**a**), VCO_2_ (**b**), and energy expenditure (**c**) and their average values from full day cycle, light cycle, and dark cycle during 48 hrs of monitoring in *Ncc*^*fl/fl*^, *Ncc*^*fl/fl*^*Ucp1*^*Cre*^ mice after 7 days of CL316243 treatment (*Ncc*^*fl/fl*^: *n* = 7; *Ncc*^*fl/fl*^*Ucp1*^*Cre*^: *n* = 6). **d**–**e** Representative images of H&E and UCP1 immunostaining of BAT sections and quantification of adipocyte size and UCP1-positive area in EAT, SAT, and BAT from indicated groups of mice (*Ncc*^*fl/fl*^*: n* = 7; *Ncc*^*fl/fl*^*Ucp1*^*Cre*^: *n* = 6). Scale: 50 μm. Inset: 25 μm. **f** RT-PCR analysis of thermogenic, lipolytic, and mitochondrial genes in EAT, SAT, and BAT from indicated mice (*Ncc*^*fl/fl*^: *n* = 7; *Ncc*^*fl/fl*^*Ucp1*^*Cre*^: *n* = 6). **g** Immunoblots and quantification of UCP1 and PGC1α relative to GAPDH in BAT from indicated mice. *n* = 6 per group. Data are mean ± SEM, two-sided Student’s t-test (**a**–**c**, **d**, **f**) and two-sided Mann-Whitney *U* test (**e**). Sample sizes were all biologically independent samples.

Impaired thermogenesis and energy expenditure in *Ncc*^*fl/fl*^*Ucp1*^*Cre*^ mice were tested by treating mice with CL316243 for 7 days. Under this treatment, *Ncc*^*fl/fl*^*Ucp1*^*Cre*^ mice showed decreased VO_2_, VCO_2_, and energy expenditure (Fig. 8a–c). H&E staining and UCP1 immunostaining revealed significantly larger lipid droplets in EAT (Fig. 8d) and smaller UCP1-positive area in SAT and BAT (Fig. 8e) from *Ncc*^*fl/fl*^*Ucp1*^*Cre*^ mice than those from *Ncc*^*fl/fl*^ mice. RT-PCR and immunoblot analyses also demonstrated decreased expression of thermogenic, lipogenic, lipolytic, and mitochondrial genes, including *Ucp1* in EAT, *Ucp1, Fas*, and *Atgl* in SAT, *Ucp1*, *Pgc1α*, *Fas*, *Atgl*, *Hsl*, *ATPase*, *Cox2*, *Cox8b*, and *Cytc* in BAT from *Ncc*^*fl/fl*^*Ucp1*^*Cre*^ mice after CL316243 treatment (Fig. 8f–g). In addition to treat mice with CL316243 for 7 days, we also housed mice under a thermoneutral temperature (30 °C) for 7 days^46^ and obtained the same observations. *Ncc*^*fl/fl*^*Ucp1*^*Cre*^ mice showed significantly lower VO_2_, VCO_2_, and energy expenditure than those of *Ncc*^*fl/fl*^ mice (Supplementary Fig. 11a–c). These observations suggest that the IL18-NCC signaling in brown and beige adipocytes mitigates obesity, insulin resistance, and adipose inflammation by maintaining the thermogenic homeostasis.

### Adipocyte-selective depletion of IL18r impairs glucose metabolism and insulin signaling

**Fig. 9:**
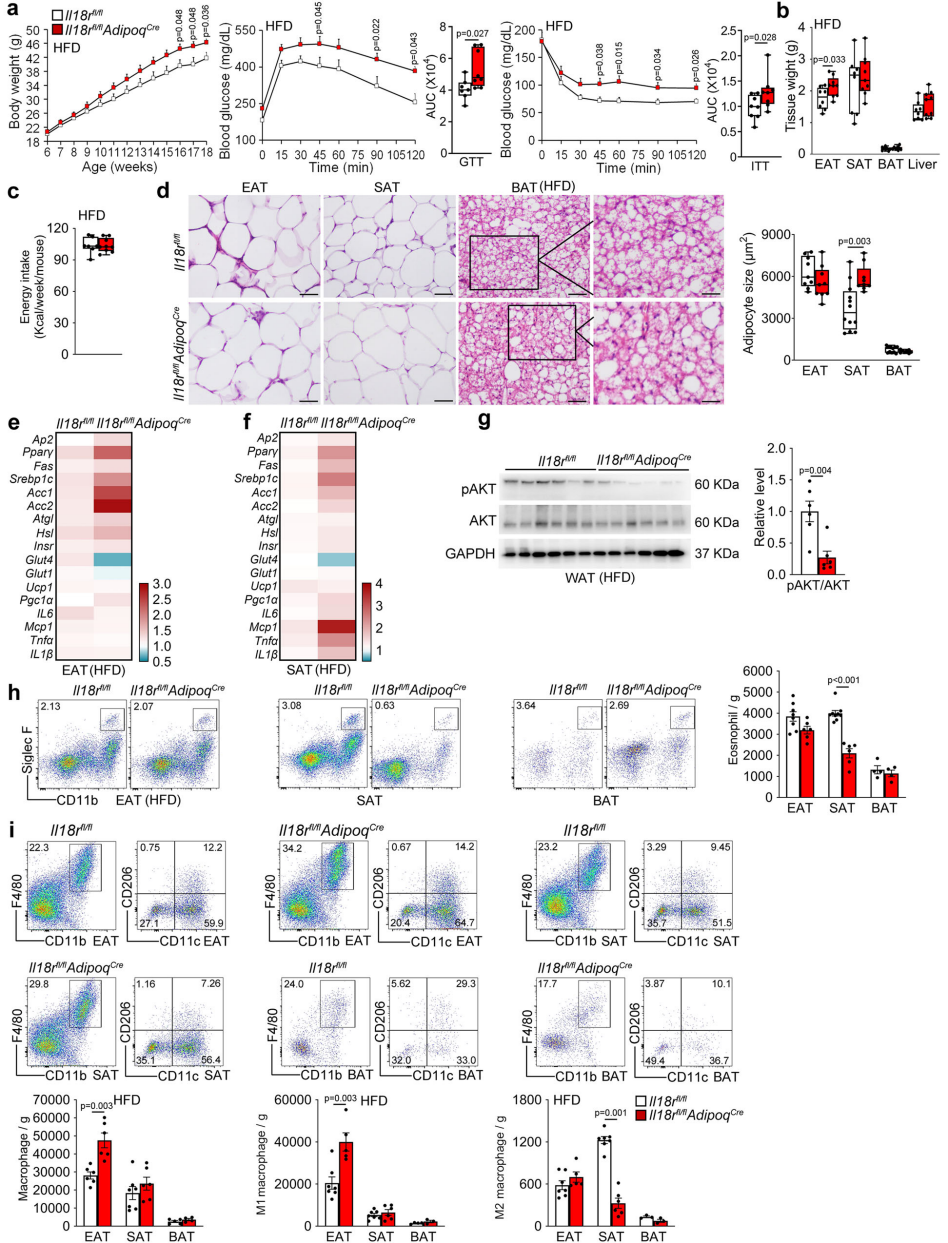
Adipocyte-selective depletion of IL18r impairs white adipocyte glucose metabolism and insulin signaling and exacerbates obesity and insulin resistance. **a**–**c** Bodyweight gain (*Il18r*^*fl/fl*^: *n* = 8; *Il18r*^*fl/fl*^*Adipoq*^*Cre*^: *n* = 9), GTT and AUC of GTT (*n* = 8 per group), ITT and AUC of ITT (*n* = 8 per group) (**a**), EAT, SAT, BAT, and liver weight (*Il18r*^*fl/fl*^*: n* = 8; *Il18r*^*fl/fl*^*Adipoq*^*Cre*^: *n* = 9) (**b**), and energy intake (*n* = 7~8 each) (*Il18r*^*fl/fl*^: *n* = 7; *Il18r*^*fl/fl*^*Adipoq*^*Cre*^: *: n* = 9) (**c**) in *Il18r*^*fl/fl*^ and *Il18r*^*fl/fl*^*Adipoq*^*Cre*^ fed a HFD for 12 weeks. **d** H&E of EAT, SAT, and BAT from indicated groups of mice (*Il18r*^*fl/fl*^-EAT: *n* = 9; *Il18r*^*fl/fl*^*Adipoq*^*Cre*^-EAT: *n* = 9; *Il18r*^*fl/fl*^-SAT: *n* = 12; *Il18r*^*fl/fl*^*Adipoq*^*Cre*^-SAT: *n* = 9; *Il18r*^*fl/fl*^-BAT: *n* = 11; *Il18r*^*fl/fl*^*Adipoq*^*Cre*^-BAT: *n* = 8). Scale: 50 μm. Inset: 25 μm. **e**–**f** RT-PCR analysis of lipogenic, lipolytic, glucose metabolic, thermogenic, and inflammatory genes in EAT (**e**) and SAT (**f**) from indicated mice (*Il18r*^*fl/fl*^: *n* = 10; *Il18r*^*fl/fl*^*Adipoq*^*Cre*^: *n* = 9). **g** Immunoblots of pAKT, AKT, and GAPDH and quantification of pAKT/AKT in WAT from indicated mice (*n* = 6 per group). **h-i**. Representative FACS images and quantification of CD11b^+^Siglec F^+^ eosinophils (*Il18r*^*fl/fl*^-EAT: *n* = 8; *Il18r*^*fl/fl*^*Adipoq*^*Cre*^-EAT: *n* = 6; *Il18r*^*fl/fl*^-SAT: *n* = 9; *Il18r*^*fl/fl*^*Adipoq*^*Cre*^-SAT: *n* = 6; *Il18r*^*fl/fl*^-BAT: *n* = 4; *Il18r*^*fl/fl*^*Adipoq*^*Cre*^-BAT: *n* = 4) (**h**) and CD11b^+^F4/80 ^+^ total, CD11b^+^F4/80 ^+^CD11c^+^ M1, and CD11b^+^F4/80 ^+^CD206^+^ M2 macrophages in EAT, SAT, and BAT (*Il18r*^*fl/fl*^-EAT: *n* = 6; *Il18r*^*fl/fl*^*Adipoq*^*Cre*^-EAT: *n* = 6; *Il18r*^*fl/fl*^-SAT: *n* = 7; *Il18r*^*fl/fl*^*Adipoq*^*Cre*^-SAT: *n* = 6; *Il18r*^*fl/fl*^-BAT: *n* = 4; *Il18r*^*fl/fl*^*Adipoq*^*Cre*^-BAT: *n* = 3) (**i**) from indicated mice. Data are mean ± SEM, two-way ANOVA repeated-measures, followed by LSD post-test (**a**), two-sided Student’s t-test (**b**–**d**, **h**) and two-sided Mann-Whitney *U* test (**g**). Sample sizes were all biologically independent samples.

To test a role for IL18r signaling from WAT adipocytes in controlling obesity and glucose intolerance, we bred the *Adipoq*^*Cre*^ mice with the *Il18r*^*fl/fl*^ mice to generate adiponectin-positive adipocyte-selective IL18r-deficient *Il18r*^*fl/fl*^*Adipoq*^*Cre*^ mice. RT-PCR verified the IL18r depletion in WAT and BAT from *Il18r*^*fl/fl*^*Adipoq*^*Cre*^ mice (Supplementary Fig. 12a). We fed *Il18r*^*fl/fl*^*Adipoq*^*Cre*^ and *Il18r*^*fl/fl*^ mice a HFD for 12 weeks to induce obesity. Compared with the *Il18r*^*fl/fl*^ mice, *Il18r*^*fl/fl*^*Adipoq*^*Cre*^ mice displayed higher bodyweight gain, glucose intolerance, and insulin resistance (Fig. 9a) and more EAT masses (Fig. 9b), although the weekly energy intake did not differ (Fig. 9c). *Il18r*^*fl/fl*^*Adipoq*^*Cre*^ also showed larger lipid droplets in SAT by H&E staining (Fig. 9d), increased lipogenic genes *Pparγ*, *Srebp1c*, *Acc1*, and *Acc2*, and decreased glucose metabolic gene *Glut4* in EAT (Fig. 9e), and increased lipogenic genes *Pparγ*, and *Srebp1c*, and inflammatory cytokines *Mcp1* and *Tnfα*, and decreased glucose metabolic gene *Glut4* in SAT (Fig. 9f) by RT-PCR, with decreased AKT activation in WAT by immunoblot analysis (Fig. 9g). However, adipocyte sizes in EAT and BAT (Fig. 9d), UCP1-positive areas in all adipose tissues (Supplementary Fig. 12b), and thermogenic genes in BAT (Supplementary Fig. 12c) did not differ between these mice. FACS analysis showed that adipocytes-selective depletion of IL18r also reduced eosinophil and M2 macrophage contents in SAT (Fig. 9h,i), and increased total and M1 macrophages in EAT (Fig. 9i). In addition, IL18r-selective depletion in adipocytes increased Th1, and decreased Th2 number in SAT from mice fed on a HFD, without affecting total CD8^+^ and CD4^+^ T cells, Th17 and Treg cells in all types of adipose tissues (Supplementary Fig. 12d–h). Eosinophils, total macrophages or M1 and M2 macrophages, total CD8^+^ and CD4^+^ T cells, Th1, Th2, Th17 and Treg cells in EAT, SAT, and spleens from *Il18r*^*fl/fl*^*Adipoq*^*Cre*^ mice did not differ from those from *Il18r*^*fl/fl*^ mice on a LFD (Supplementary Fig. 13a–e). Together, these observations suggest that IL18-IL18r signaling in WAT adipocytes protects mice from obesity, insulin resistance and adipose inflammation by maintaining the homeostatic glucose metabolism and insulin signaling.

## Discussion

**Fig. 10:**
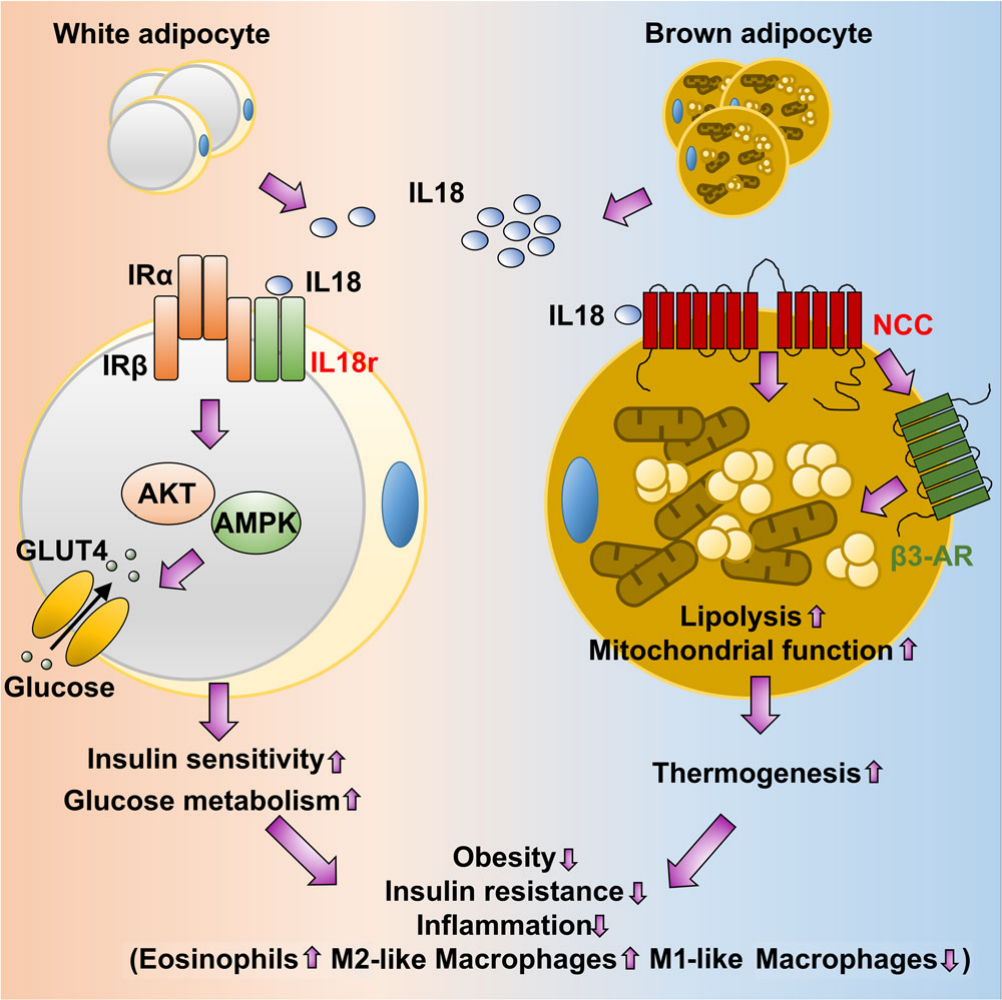
The proposed mechanisms for IL18 signaling-regulated thermogenesis and glucose metabolism in adipose tissues. IL18 is an adipokine from brown adipocytes in BAT and beige cells in SAT. NCC mediates IL18 functions in activating thermogenesis by regulating mitochondrial functions and lipolysis in BAT in cooperation with β3-AR. IL18r mediates IL18 activities in promoting glucose tolerance and insulin sensitivity by regulating IR-AKT insulin signaling and GLUT4-mediated glucose metabolism in EAT. IL18 signaling in adipocytes protects mice from obesity, insulin resistance and adipose tissue inflammation.

IL18 activities in obesity and diabetes have been reported but studies remain observational and controversial^28–31,34^. Paradoxical observations suggest the complexity of IL18 functions in adipose tissue energy metabolism. For example, after long-term cold exposure, UCP1 expression in BAT did not differ among WT, *Il18*^*−/−*^, and *Il18r*^*−/−*^ mice, but UCP1 expression was significantly reduced in iWAT from *Il18*^*−/−*^ mice and increased in iWAT from *Il18r*^*−/−*^ mice^29^, suggesting that different mechanisms regulate the IL18 functions in BAT and iWAT. Recent discovery of an alternative IL18 receptor NCC helped address many of these similar paradoxical observations regarding the pro-inflammatory functions of IL18 in cardiovascular diseases^35^. As we summarized in Fig. 10, here we report the beneficial but divergent roles of IL18 in thermogenesis and in glucose metabolism and insulin sensitivity, although both activities of IL18 led to the same reduced obesity, glucose intolerance, and insulin resistance in mice on a HFD. These functions of IL18 are mediated by IL18r and NCC in different adipose tissues. NCC dominantly regulates the role of IL18 in thermogenesis in UCP1-positive brown and beige adipocytes, whereas IL18r mediates the role of IL18 in glucose metabolism and insulin signaling in adiponectin-expressing adipocytes. These findings establish a regulatory link between inflammatory signaling and adipocyte metabolism and suggest that the IL18 signaling from NCC and IL18r in different adipose tissues (Fig. 10) represents distinct therapeutic approaches to protect against thermogenesis-defect-associated energy expenditure and obesity and insulin signaling defect-associated diabetes.

The relationships among the immune system, adipocytes, and systemic metabolism can be sophisticated. Prior studies showed paradoxical increases in obesity and insulin resistance, and decrease in thermogenesis in mice deficient in pro-inflammatory IL6^47,48^, and development of obesity and metabolic syndrome upon depletion of anti-inflammatory IL10^14^. IL18 accelerates atherogenesis by binding to NCC and IL18r on vascular smooth muscle cells, endothelial cells, and macrophages^35,49^. Yet, IL18 reduces bodyweight gain and insulin resistance and increases energy homeostasis^25,28,31^. The roles of IL18 in cardiovascular diseases and metabolic diseases are fully opposite. NCC inhibitor hydrochlorothiazide reduces atherosclerotic lesion growth and lesion inflammatory cell contents in *Apoe*^*−/−*^*Il18r*^*−/−*^ mice^35^. Yet clinical studies showed that the same NCC inhibitor contributes to the comorbidities of obesity and metabolic syndrome. These clinical observations have remained mysterious^50–52^. Results from our study suggest that these adverse effects from hydrochlorothiazide-treated patients are associated with IL18 signaling defect in brown and beige adipocytes.

The same cytokine pathways may exert different pathophysiological effects depending on the source of the cytokine, the duration of the exposure, and the responding cell types. We proposed the possibilities that IL18 activates the insulin signaling via the interaction between IL18r and IRβ in white adipocytes and activates PGC1α and mitochondrial respiration primarily through the NCC in brown and beige adipocytes (Fig. 10). Previous reports showed that IL18 activates the toll-like receptor signaling cascade, leading to the activation of the NF-κB pathway and the production of inflammatory cytokines^53^. IL18 also activates MAP kinase, PI3-kinase, and STAT3 pathways^54^. IL18 enhances AMPK signaling and lipid oxidation in skeletal muscle^34^. These pathways may also contribute to the metabolic consequences of IL18r or/and NCC depletions. PPARγ is an adipocyte-predominant transcription factor that regulates adipogenesis, lipid metabolism, cell proliferation, inflammation and insulin sensitization^55^. Both forms of PPARγ1 and PPARγ2 are essential for the development of adiposity and control of insulin sensitivity^56^. In this study, we demonstrated that PPARγ2 expression was decreased in BAT from HFD-fed IL18r or NCC single deficient mice, or combined deficient mice. It is possible that mutation of the IL18 signaling decreased the expression or activity of PPARγ2, thereby suppressing the mitochondrial respiration and lipolysis in BAT, a hypothesis that has not been explored in this study. It was reported that mice lacking the NLRP1 inflammasome showed reduced VAT IL18 production and lipolysis, leading to obesity and metabolic syndrome. NLRP1 activation increased plasma IL18 levels, decreased adiposity. And mice were resistant to diet-induced metabolic dysfunction^57^. Here, we showed that BAT-selective depletion of IL18 or NCC reduced BAT thermogenesis and lipolysis, and increased HFD-induced obesity and metabolic syndrome. In vitro, IL18 elevated the brown adipocyte extracellular oxygen consumption rate (OCR) and thermogenesis gene expression by activating isoproterenol-stimulated uncoupled respiration and lipolysis.

Deficiency of IL18 or IL18r in mice leads to hyperphagia^28,30,31^. We obtained similar observations. Global deficiency of IL18r or combined deficiency of NCC and IL18r increased mouse energy intake. Consistent with this observation, BAT-selective depletion of IL18 also increased mouse energy intake. Surprisingly, BAT-selective NCC depletion or WAT-selective depletion of IL18r did not affect mouse energy intake. These observations suggest that BAT-derived IL18 is essential to energy intake, but this activity of IL18 does not involve NCC or IL18r in adipose tissues. It is possible that BAT-derived IL18 is functionally linked to the hypothalamus as part of the interactions with the central nervous system (CNS) to maintain the metabolic homeostasis. Changes of IL18 expression in the periphery BAT may provide a feed-back signal to the CNS to control body energy expenditure or storage as leptin does^58^. The existence of such a loop between the hypothalamic neuropeptide Y and BAT-derived IL18 in response to obesity and thermogenic activation is plausible and merits further investigation.

Together, the observations from this study suggest that IL18 is a brown adipokine that is sensitive to thermogenic activation and HFD feeding. IL18 signaling with its two receptors NCC and IL18r is required for the maintenance of adipose tissue homeostasis. IL18 promotes BAT thermogenesis predominantly through NCC and maintains glucose sensitivity and insulin signaling through IL18r, thereby mitigating metabolic disorders. These findings not only reinforce the role of IL18 axes in regulating thermogenesis and insulin sensitivity but also advance our understanding the complexity of the link between immune signaling and adipose tissue function. Target of IL18 axis activity may become an attractive therapeutic strategy for obesity and diabetes.

## Methods

All animal procedures conformed to the Guide for the Care and Use of Laboratory Animals published by the US National Institutes of Health and was approved by the Brigham and Women’s Hospital Standing Committee on Animals (protocol #2016N000442).

Use of discarded and de-identified human adipose tissues (obtained from the tissue bank of the Department of Surgery, Brigham and Women’s Hospital), was approved by the Partners Human Research Committee, Protocol# 2010P001930.

### Mice and indirect calorimetry

WT (000664, C57BL/6, The Jackson Laboratory, Bar Harbor, ME), *Il18r*^*−/−*^ (004131, C57BL/6.129, The Jackson Laboratory), and *Ncc*^*−/−*^ mice (C57BL/6)^35^ were crossbred to generate the WT, *Il18r*^*−/−*^, *Ncc*^*−/−*^, and *Ncc*^*−/−*^*Il18r*^*−/−*^ mice. *Ucp1*^*Cre*^ mice (024670, C57BL/6, The Jackson Laboratory) and *Il18*^*fl/fl*^ mice^59^ or *Ncc*^*fl/fl*^ (C57BL/6, produced from Cyagen Biosciences Inc, Santa Clara, CA) were crossbred to generate *Il18*^*fl/fl*^*Ucp1*^*Cre*^ and *Il18*^*fl/fl*^ control mice or *Ncc*^*fl/fl*^*Ucp1*^*Cre*^ and *Ncc*^*fl/fl*^ control mice. *Il18r*^*fl/fl*^ mice^59,60^ were crossed with the *Adipoq*^*Cre*^ mice (028020, C57BL/6, The Jackson Laboratory) to generate the *Il18r*^*fl/fl*^*Adipoq*^*Cre*^ and *Il18r*^*fl/fl*^ control mice. All mice had the same genetic background. All mouse experiments were performed using their male littermates based on their availability from crossbreeding. Mice were housed in ventilated cages in a pathogen-free barrier facility that was maintained at 22 ± 2 °C and with a 12-h light-12-h dark cycle, and free to access autoclaved water and irradiated food throughout the study. Six-week-old male mice consumed a HFD (D12492, Research Diets Inc., New Brunswick, NJ) for 12 weeks to develop obesity and insulin resistance. Mouse bodyweight and energy intake was monitored weekly. Mice at 12-week-old were i.p. injected with or without 1 mg/day/kg CL316243 (1499, TOCRIS, Minneapolis, MN), a β3-AR agonist, for 7 days, 4 °C exposure for 7 days, or housed at thermoneutral temperature 30 °C for 7 days. For indirect calorimetry, mice were acclimated to a Columbus Instruments Oxymax Comprehensive Lab Animal Monitoring System (CLAMS) for 2 days. O_2_ consumption (VO_2_), CO_2_ production (VCO_2_), and energy expenditure (EE: [VO_2_*CVO_2_ + VCO_2_*CVCO_2_]/1000) were continuously monitored during the next 48 hrs at ambient room temperature (21–23 °C). CVO_2_ and CVCO_2_ are thermal equivalent of O_2_ and CO_2_, and preset parameter as 3.9410 and 1.1060 in CLAMS. We used CalR (https://calrapp.org/) to analysis VO_2_, VCO_2_, and EE followed the most current analysis protocols. After 12 weeks on an HFD or metabolic rate measurement, mice were euthanized by CO_2_, and the plasma, liver, and adipose tissue were collected, including EAT, SAT, and BAT.

### Glucose tolerance test (GTT) and insulin tolerance test (ITT)

Mouse GTT was performed after 16 hrs of fasting and ITT was performed after 4 hrs of fasting. In brief, mice were i.p. injected with D-glucose (1 g/kg bodyweight, G7021, Sigma-Aldrich, St. Louis, MO) for GTT or with insulin (1.5 IU/kg bodyweight, NDC 0169-1833-11, NOVOLIN, Wellington, FL) for ITT. Blood glucose levels were measured from tail veins using a blood glucose meter (Bayer Healthcare LLC, Mishawaka, IN) at 0, 15, 30, 45, 60, 90, and 120 min after injection.

### Histology analysis

Adipose tissues were fixed in 4% formalin, embedded in paraffin, and serially sliced into 6 μm thickness. For histological characterizations, adipose tissue paraffin sections were used for hematoxylin (GHS316, Sigma-Aldrich) & eosin (HT110116, Sigma-Aldrich) staining. Five random fields from each section were examined, and adipocyte average area was quantified using the Image-Pro Plus software (Media Cybernetics, Bethesda, MD).

For immunohistochemical analysis, mouse paraffin sections were deparaffinized, rehydrated, and incubated with the following primary antibodies: rabbit anti-IL18, goat anti-IL18r, rabbit anti-NCC, mouse anti-UCP1, and rat anti-Mac-3, followed by appropriate biotin-conjugated secondary antibodies and HRP-streptavidin. All antibody dilution, catalog number, and vendor information are listed in Supplementary Table 1. After detection with AEC chromogenic agent (K3464, DAKO), slides were counterstained with hematoxylin. Representative images were acquired with a Leica DM 1000 LED microscope. The UCP1 and Mac-3-positive areas in five random fields in each section were determined by detecting the staining intensity with Image-Pro Plus and data were presented as positive area percent.

For immunofluorescent co-localization analyses, adipose tissue paraffin sections were sequentially stained with rabbit anti-IL18 and mouse anti-UCP1 antibodies, or goat anti-IL18r and mouse anti-IRβ antibodies, followed by Alex Fluor 555- or 488-labeled secondary antibody detections. Slides were counterstained with DAPI (R37606, Invitrogen, Carlsbad, CA), and images captured with a confocal microscopy (Olymbus Fluoview FV1000; Olymbus).

### Immunoblot analysis and immunoprecipitation

For immunoblot analysis, an equal amount of proteins extracted from different adipose tissues or primary cells were separated on SDS-PAGE, blotted, and detected with the following primary antibodies: rabbit anti-IL18, goat anti-IL18r, rabbit anti-NCC, mouse anti-UCP1, rabbit anti-PGC1α, rabbit anti-ATGL, rabbit anti-β3-AR, goat anti-GLUT4 and mouse anti-PPARγ, and rabbit anti-pAMPK, rabbit anti-AMPK, rabbit anti-pHSL, rabbit anti-HSL, rabbit anti-COX IV, rabbit anti-Cyt C, rabbit anti-pAKT, rabbit anti-AKT, rabbit anti-pIRβ, mouse anti-IRβ, rabbit anti-pPKA, rabbit anti-PKA, rabbit anti-GAPDH, and rabbit anti-β-actin antibodies, followed by appropriate HRP-conjugated secondary antibodies. All antibody dilution, catalog number, and vendor information are listed in Supplementary Table 1.

For immunoprecipitation, primary cultured epididymal pre-adipocytes were isolated and differentiated to mature adipocytes. Cells were lysed in an immunoprecipitation lysis buffer (0.025 M Tris, 0.15 M NaCl, 0.001 M EDTA, 1% NP-40, 5% glycerol; pH 7.4). Equal amounts of cell lysates (0.25 mg) were subsequently incubated overnight at 4 °C with goat anti-IL18r antibody (10 μg, AF587, R&D Systems), rabbit anti-NCC antibody (10 μg, AB3553, Millipore), or IgG isotype control antibody (goat IgG isotype control antibody, 10 μg, 02-6202, Thermo Fisher Scientific), or rabbit IgG isotype control antibody (10 μg, 02-6102, Thermo Fisher Scientific). The antibody-antigen complexes were captured, washed and eluted according to the manufacturer’s instructions (26149, Thermo Fisher Scientific). Immunoprecipitates were then resolved in SDS under reducing condition with 5 µg input protein of the whole cell lysate, and followed by immunoblotting with mouse anti-IRβ antibody (1:1000, 3020, Cell Signaling Technology) to detect the immunocomplexes.

### Real-time PCR

Total RNA was extracted from adipose tissues or primary cells using the Trizol reagent (15596018, Invitrogen), and then reverse transcribed to cDNA using the high-capacity cDNA reverse transcriptase (4368813, Invitrogen) according to manufacturer’s protocol. Real-time PCR was analyzed using SYBR green dye (1725125, Bio-Rad, Hercules, CA) in a Bio-Rad MyiQ2 Real-time PCR System. Data were processed using the ∆∆CT method when GAPDH was used as the reference gene. All primer sequences used in this study were listed in the Supplementary Table 2.

### Plasma insulin, leptin, and inflammatory cytokines measurement

The plasma levels of IL18 (BMS618-3, Invitrogen), insulin (90080, Crystal Chem, Elk Grove Village, IL), leptin (RD291001200R, BioVendor, Asheville, NC), IL1β (88-7013-22), TNF-α (88-7324-22), IL6 (88-7064-88), MCP1 (88-7391-88), IFN-γ (88-7314-88) from Invitrogen were assessed using the mouse ELISA kits according to the manufacturers.

### Cell culture

Primary brown and white pre-adipocytes were fractionated according to our previous report^60^. Briefly, adipose tissues were dissected, minced and digested with collagenase D (11088882001, Sigma-Aldrich) for 25 min at 37 °C. The digested tissues were filtered through a 100-μm mesh filter and centrifuged at 200 g for 10 min at 4 °C. The cell pellets were suspended with red blood cell lysis buffer for 5 min. After centrifugation, the fractionated pre-adipocytes were cultured in Dulbecco’s modified Eagle’s medium/F-12 (11320033, Thermo Fisher Scientific) supplemented with 10% fetal bovine serum (FBS). For browning differentiation, confluent cells were induced in pre-adipocyte cultures containing 0.5 mM isobutylmethylxanthine (I7018), 125 nM indomethacin (I7378), 1 μM dexamethasone (D4902), 850 nM insulin (I2643), 1 nM 3,3′,5-triiodo-L-thyronine (T3, T2877) and 1 μM rosiglitazone (R2408) from Sigma-Aldrich for 48 hrs. Cells were then maintained in a medium with 850 nM insulin, 1 nM T3, and 1 μM rosiglitazone for 6 days. After differentiation, brown adipocytes were starved for 8 hrs, followed by treating cells with 10 or 100 ng/mL IL18 (B004-2, MBL International, Woburn, MA) for 24 h. For lipolysis induction, cells were treated with 3 µM isoproterenol (I6504, Sigma-Aldrich) for 3 hrs. For white adipocyte differentiation, confluent cells were induced in pre-adipocyte cultures containing 0.5 mM isobutylmethylxanthine, 1 μM dexamethasone, and 850 nM insulin for 48 hrs. The cells were then maintained in a medium containing 850 nM insulin for 6 days. After differentiation, the cells are stained with oil red O and quantified lipid accumulation by absorbance at OD 510 nm. After differentiation, white adipocytes were starved for 8 hrs, followed by treating cells with 10 or 100 ng/mL IL18 for 24 hrs. For insulin signaling activation, cells were starved for 6 hrs, followed by treating cells with 10 or 100 ng/mL IL18 with or without 20 nM insulin for 30 min. For cAMP activation, cells were starved for 8 hrs, followed by treating cells with 100 ng/mL IL18 with or without 20 µM H89 (9844 S, Cell Signaling Technology) for 30 min.

Human adipose tissue from two donors were used in this study to isolate human pre-adipocytes. Donor information is listed in Supplementary Table 3. Human pre-adipocytes were isolated from obese subcutaneous adipose tissue and induced for browning differentiation the same as mouse pre-adipocytes. After differentiation, brown human adipocytes were starved for 8 hrs, followed by treating the cells with 0, 10 or 100 ng/mL recombinant human IL18 (9124-IL-050, R&D Systems) for 24 h.

### Flow cytometry

SVFs were isolated by collagenase digestion from EAT, SAT, and BAT as described above, and resuspended in FACS buffer after red blood cell lysis. After 15 min incubation with an Fc block (16-0161-82; eBioscience, San Diego, CA), SVFs cells were stained with FACS antibodies for 30 min at 4 °C. To assess total macrophage, M1 macrophage, and M2 macrophage number in SVFs, cells were stained with Fixable Viability Dye eFluor 450, anti-CD45-APC, anti-CD11b-APC-Cyanine7 and anti-F4/80-PE, anti-CD206-PerCP-Cyanine5.5 and anti-CD11c-FITC. To detect eosinophil number in SVFs, cells were stained with Fixable Viability Dye eFluor 450, anti-CD45-PerCP-Cyanine5.5, anti-CD11b-APC, and anti-Siglec-F-PE antibodies. To detect CD4^+^ and CD8 T^+^ cell number in SVFs, cells were stained with Fixable Viability Dye eFluor 450, anti-CD45-PerCP-Cyanine5.5, anti-CD4-FITC, and anti-CD8-PE antibodies. To detect Treg cell number in SVFs, cells were stained with Fixable Viability Dye eFluor 450, anti-CD45-PerCP-Cyanine5.5, anti-CD4-FITC, anti-CD3-APC- Cyanine7, and anti-CD25-PE antibodies. Cells were fixed, permeabilized, and intracellularly stained for anti-Foxp3-APC antibodies. To detect Th1, Th2, Th17 cell number in SVFs, the cells were activated with phorbol myristate acetate (PMA, 50 ng/ml, 16561-29-8, Sigma) and ionomycin (1 µg/mL, 10634, Sigma) for 5 hrs in the presence of monensin (2 μM, 420701, BioLegend) at the end 3 hrs at 37 °C in a 5% CO_2_ atmosphere. After activation, cells were stained with Fixable Viability Dye eFluor 450, anti-CD45-PerCP-Cyanine5.5, anti-CD3-APC- Cyanine7, anti-CD4-FITC antibodies. Cells were fixed, permeabilized, and intracellularly stained for anti-IFN-γ-PE, anti-IL4-APC, and anti-IL17A-PE-Cyanine7 antibodies. Cells were initially selected by size on the basis of forward scatter (FSC) and side scatter (SSC), following separated on the basis of cell-surface markers using a FACS Analyzer LSR (BD Biosciences). Antibody dilution and vendor information are listed in Supplementary Table 1.

### Extracellular respiration

Primary brown adipocytes from different groups of mice were plated in seahorse plate and induced to mature brown adipocytes as described above. After differentiation, brown adipocytes were starved for 8 hrs, followed by treating the cells with or without 100 ng/mL IL18 for 24 h. Oxygen consumption rate (OCR) was performed on fully differentiated brown adipocytes (at day 8 of differentiation) with the Agilent Seahorse XF96 cellular Flux Analyzer (Agilent Technologies, Inc., Santa Clara, CA). Mitochondrial respiration was quantified using the Mito-stress test protocol. Four measurements were obtained under basal conditions and upon addition of isoproterenol (1 µM, 10009951, Cayman Chemical, Ann Arbor, MI), oligomycin (1 µg/mL, 75351), fluoro-carbonyl cyanide phenylhydrazone (FCCP, 1 µg/mL, C2920), rotenone (3 µM, R8875), and antimycin A (2 µg/mL, A8674) from Sigma-Aldrich. Non-mitochondrial respiration was subtracted to obtain basal, basal uncoupled, isoproterenol-stimulated uncoupled and maximal mitochondrial respiration.

### In vitro glucose uptake assay

Primary white or brown adipocytes from different groups of mice were plated on a 96-well black with clear bottom plate. After differentiation, white or brown adipocytes were starved for 8 hrs, followed by treating the cells with or without 20 nM insulin and/or 100 ng/mL IL18 for 24 hrs. To determine the 2-[N-(7-nitrobenz-2-oxa-1,3-diazol-4-yl) amino]-2-deoxy-D-glucose (2-NBDG) uptake level, cells were treated with glucose-free culture medium for 10 min and followed by treated with 100 µg/mL 2-NBDG in glucose-free medium for 2 hrs. 2-NBDG was assessed using the Glucose Uptake Cell-Based Assay Kit (600470, Cayman Chemical) according to manufacturers.

### In vitro cAMP assay

Differentiated brown adipocytes from different groups of mice were starved for 8 hrs, followed by treating the cells with or without 100 ng/mL IL18 and 20 µM H89 for 24 hrs. Cell were extraction with 0.1 M HCl and centrifuged at 1000 g for 10 min. cAMP level was determined using the cAMP ELISA Kit (581001, Cayman Chemical) according to manufacturers.

### Lipolysis

Differentiated mature adipocytes were chased with DMEM/F-12 containing 2% fatty acid-free bovina serum albumin (BSA) and treated with 3 mM isoproterenol for 3 hrs. The glycerol levels from the supernatants were quantified using a commercial kit (10009381, Cayman Chemical). The glycerol amounts were normalized to the total protein content of the differentiated adipocytes using a Pierce BCA protein assay reagent (PI23223, Thermo Fisher Scientific).

### Mitochondrial nuclear DNA quantification

Total DNA from BAT was extracted using the DNeasy blood and tissue kit (96506, Qiagen, Germantown, MD), and the relative levels of mitochondrial DNA and nuclear DNA were quantified using primers specific for mitochondrial 16S rRNA and nuclear 18S rRNA genes. RT-PCR primers are listed in Supplementary Table 2.

### Statistics

All data were expressed as mean ± SEM. Non-parametric Mann-Whitney *U* test was used for data comparisons between two groups with abnormal data distribution. Unpaired two-tailed Student’s t-test was used for two group data comparisons when data passed normality and equal variance test. One-way ANOVA test was used for data analyses with multiple group comparisons. The statistical significance of weekly bodyweight gain, GTT, and ITT was determined with a two-way ANOVA repeated measure. LSD post-hoc tests was used for multiple test correction of ANOVA to obtain the *P* value for the bracketed pairwise comparison. GraphPad Prism 9 version was used for analysis and *P* < 0.05 were considered significant.

### Reporting summary

Further information on research design is available in the Nature Portfolio Reporting Summary linked to this article.

## Supplementary information

Supplementary Information

Reporting Summary

## Data Availability

Data supporting the findings of this manuscript are available from the corresponding authors upon reasonable request. The source data underlying all Figures and Supplementary Figures are provided as a Source Data file. Source data are provided with this paper.

## Source data

Source Data
