# Supplementary material for: Differential IL18 signaling via IL18 receptor and Na-Cl co-transporter discriminating thermogenesis and glucose metabolism regulation
Source: Nat Commun. 2022 Dec 8;13:7582. doi: 10.1038/s41467-022-35256-8 (PMC9732325; doi:10.1038/s41467-022-35256-8)
Supplement: Supplementary file 1 — Supplementary Information [file 41467_2022_35256_MOESM1_ESM.pdf]

Supplementary Materials

Differential IL18 signaling *via* IL18 receptor and Na-Cl co-transporter discriminating thermogenesis and glucose metabolism regulation

Xian Zhang,[1,2] Songyuan Luo,[2,3] Minjie Wang,[2] Qiongqiong Cao,[1] Zhixin Zhang,[1] Qin Huang,[2] Jie Li,[2] Zhiyong Deng,[2] Tianxiao Liu,[2] Cong-Lin Liu,[2,4] Mathilde Meppen,[2] Amelie Vromman,[2] Richard A. Flavell,[5] Gökhan S. Hotamışlıgil,[6] Jian Liu,[1] Peter Libby,[2] Zhangsuo Liu,[4] Guo-Ping Shi[2]

1) School of Food and Biological Engineering, Hefei University of Technology, Hefei, Anhui 230009, China
2) Department of Medicine, Brigham and Women's Hospital and Harvard Medical School, Boston, MA 02115, USA
3) Department of Cardiology, Guangdong Cardiovascular Institute, Guangdong Provincial People's Hospital, Guangdong Academy of Medical Sciences, Guangzhou, Guangdong 510000, China
4) Department of Nephrology, the First Affiliated Hospital, Research Institute of Nephrology, Zhengzhou University, Henan Province Research Center For Kidney Disease, Key Laboratory of Precision Diagnosis and Treatment for Chronic Kidney Disease in Henan Province, Zhengzhou, Henan 450052, China
5) Department of Immunobiology, Yale School of Medicine, New Haven, CT 06520, USA
6) Department of Molecular Metabolism, Harvard School of Public Health, Boston, MA 02115, USA

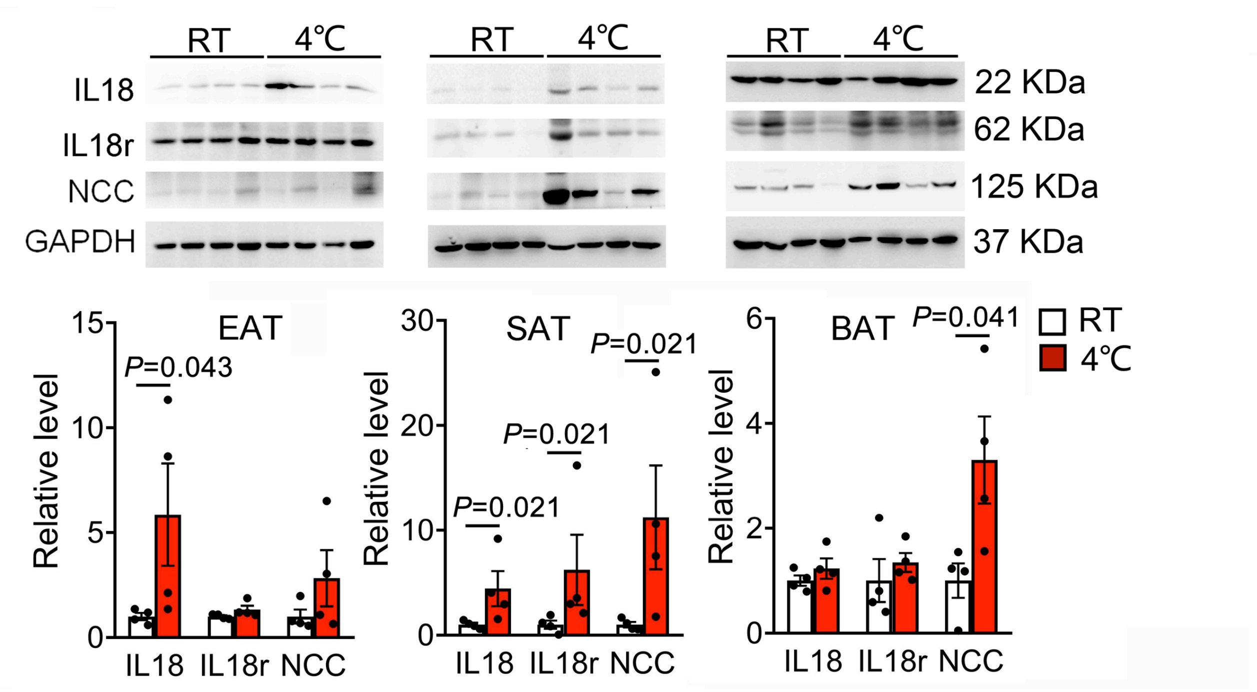

Supplementary Fig. 1. Adipose tissue expressions of IL18, IL18r, and NCC in EAT, SAT, and BAT from WT mice in response to thermogenic activation at room temperature (RT) or 4 ^{o}C for 7 days on a LFD (n=4 biologically independent samples per group). Data are mean±SEM, two-sided Mann-Whitney *U* test.

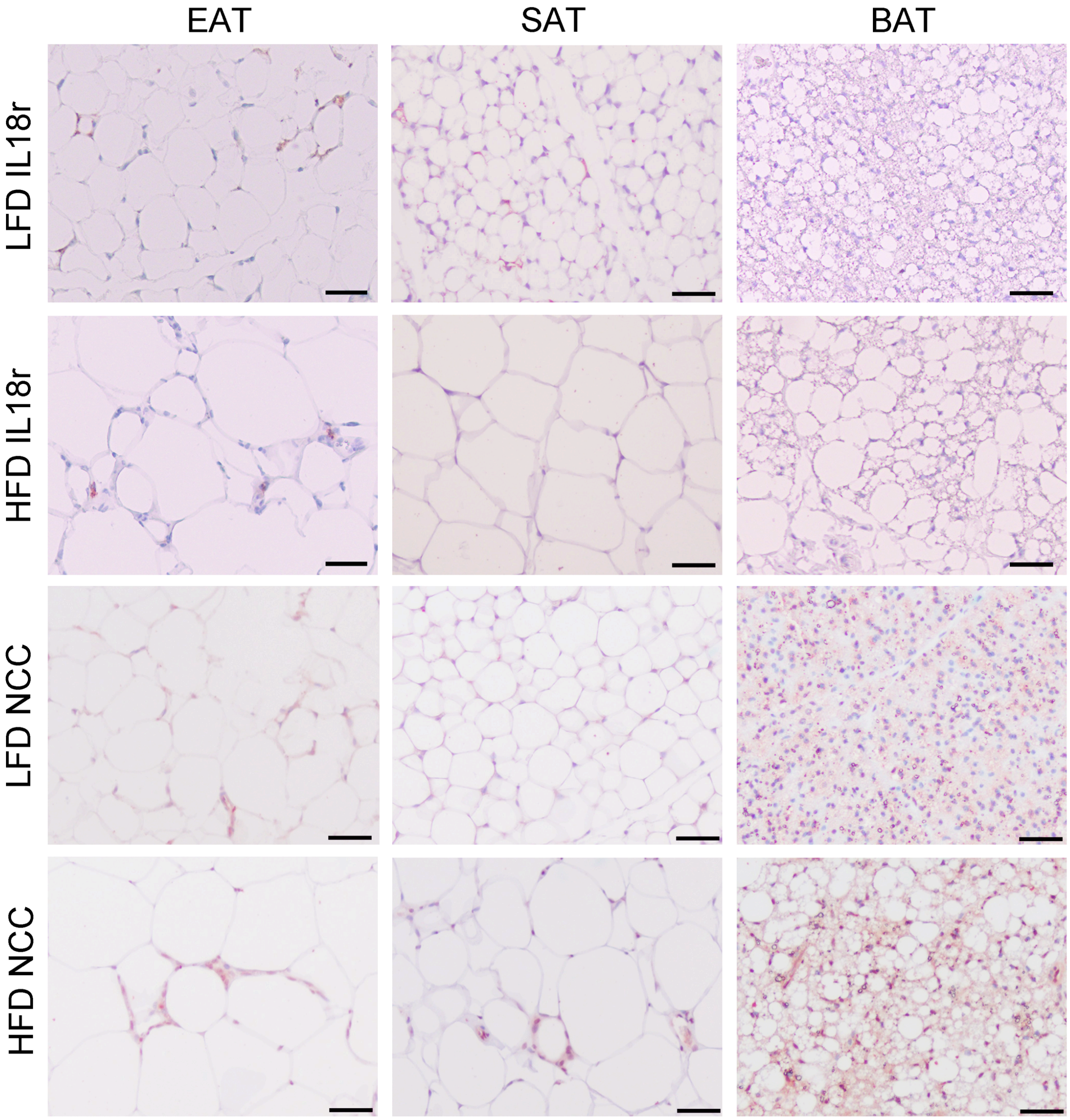

Supplementary Fig. 2. IL18r and NCC expression in different types of adipose tissues. Representative immunostaining of NCC and IL18r in EAT, SAT and BAT sections from WT mice on a LFD or HFD (n=8 biologically independent samples per group). Scale: 50 μm.

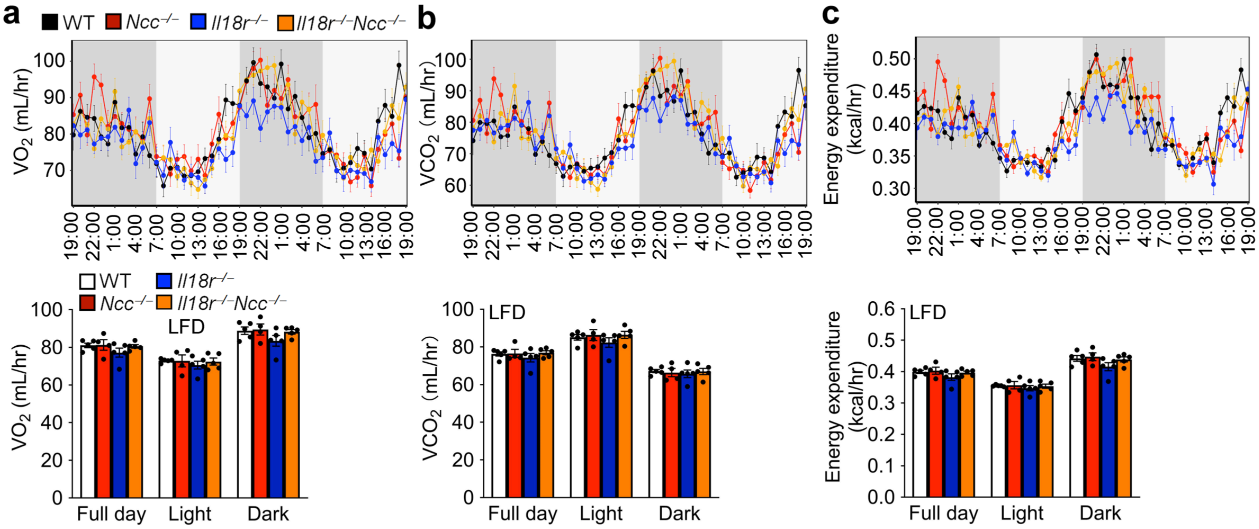

Supplementary Fig. 3. Energy expenditure in LFD-fed mice without CL316243 treatment. **a-c.** Mouse metabolic parameters, including oxygen consumption (VO_2) (**a**), carbon dioxide production (VCO_2) (**b**), and energy expenditure (**c**) and their average values from full day cycle, light cycle, and dark cycle during 48 hrs of monitoring in LFD-fed WT, *Ncc*$^{-/-}$, *Il18r*$^{-/-}$, and *Il18r*$^{-/-}$*Ncc*$^{-/-}$ mice (WT: n=5; *Ncc*$^{-/-}$: n=4, *Il18r*$^{-/-}$: n=5; *Il18r*$^{-/-}$*Ncc*$^{-/-}$: n=5 biologically independent animals). Data are mean±SEM. One-way ANOVA test, followed by LSD post-test.

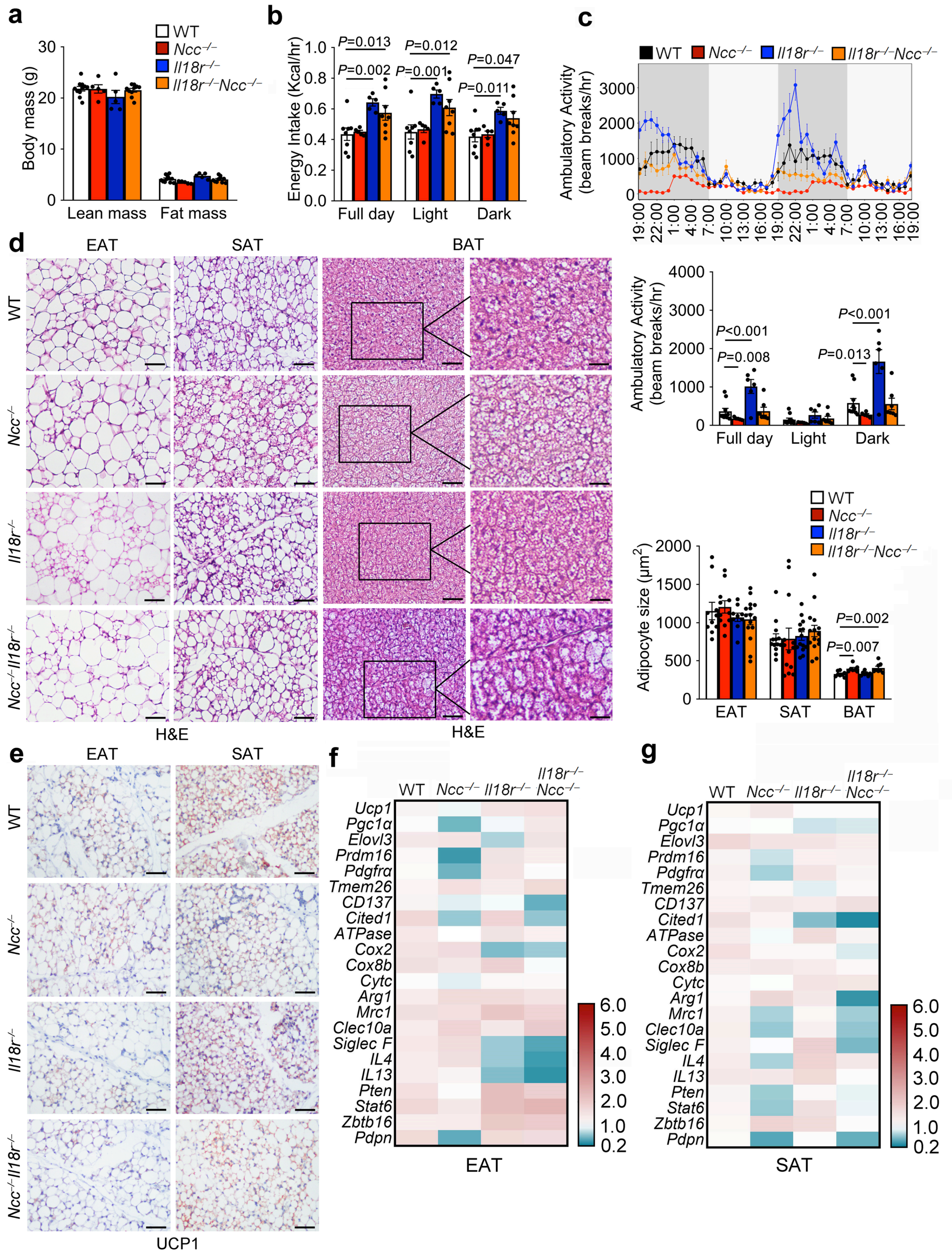

a
Body mass (g)
WT
Ncc−/−
Il18r−/−
Il18r−/−Ncc−/−
Lean mass
Fat mass
b
Energy Intake (Kcal/hr)
P=0.013
P=0.002
P=0.012
P=0.001
P=0.047
P=0.011
Full day
Light
Dark
c
Ambulatory Activity (beam breaks/hr)
P<0.001
P=0.008
P=0.013
P<0.001
d
EAT
SAT
BAT
H&E
Adipocyte size (μm²)
P=0.002
P=0.007
e
UCP1
f
EAT
g
SAT
Ucp1
Pgc1α
Elovl3
Prdm16
Pdgfrα
Tmem26
CD137
Cited1
ATPase
Cox2
Cox8b
Cytc
Arg1
Mrc1
Clec10a
Siglec F
IL4
IL13
Pten
Stat6
Zbtb16
Pdpn

Supplementary Fig. 4. Bodyweight, adipocyte morphology, and gene expressions in WT, *Ncc*$^{-/-}$, *Il18r*$^{-/-}$ and *Il18r*$^{-/-}$*Ncc*$^{-/-}$ mice in response to CL316243. **a-c**. Body mass (WT: n=11; *Ncc*$^{-/-}$: n=5, *Il18r*$^{-/-}$: n=5; *Il18r*$^{-/-}$*Ncc*$^{-/-}$: n=10 biologically independent animals) (**a**), food intake during the day-night cycles (WT: n=8; *Ncc*$^{-/-}$: n=5, *Il18r*$^{-/-}$: n=5; *Il18r*$^{-/-}$*Ncc*$^{-/-}$: n=8 biologically independent animals) (**b**), and ambulatory activity and their average values (WT: n=10; *Ncc*$^{-/-}$: n=5, *Il18r*$^{-/-}$: n=6; *Il18r*$^{-/-}$*Ncc*$^{-/-}$: n=7 biologically independent animals) (**c**). **d**. Representative images of H&E staining and quantification of adipocyte sizes in EAT, SAT, and BAT (WT-EAT: n=10; *Ncc*$^{-/-}$-EAT: n=11, *Il18r*$^{-/-}$-EAT: n=9; *Il18r*$^{-/-}$*Ncc*$^{-/-}$-EAT: n=15; WT-SAT: n=13; *Ncc*$^{-/-}$-SAT: n=13, *Il18r*$^{-/-}$-SAT: n=14; *Il18r*$^{-/-}$*Ncc*$^{-/-}$-SAT: n=15; WT-BAT: n=8; *Ncc*$^{-/-}$-BAT: n=7, *Il18r*$^{-/-}$-BAT: n=7; *Il18r*$^{-/-}$*Ncc*$^{-/-}$-BAT: n=8 biologically independent samples). **e**. Representative images of UCP1 immunostaining (n=7~10/group) of EAT and SAT. Scale: 50 μm, inset: 25 μm. **f-g**. RT-PCR analysis of thermogenic, mitochondrial, lipogenic, and lipolytic genes, and M2 macrophage markers, eosinophil marker, type 2 cytokines, and Treg functional molecules in EAT (WT: n=7; *Ncc*$^{-/-}$: n=6, *Il18r*$^{-/-}$: n=7; *Il18r*$^{-/-}$*Ncc*$^{-/-}$: n=8 biologically independent samples) (**f**) and SAT (WT: n=7; *Ncc*$^{-/-}$: n=6, *Il18r*$^{-/-}$: n=7; *Il18r*$^{-/-}$*Ncc*$^{-/-}$: n=9 biologically independent samples) (**g**). Data are mean±SEM, one-way ANOVA test, followed by LSD post-test.

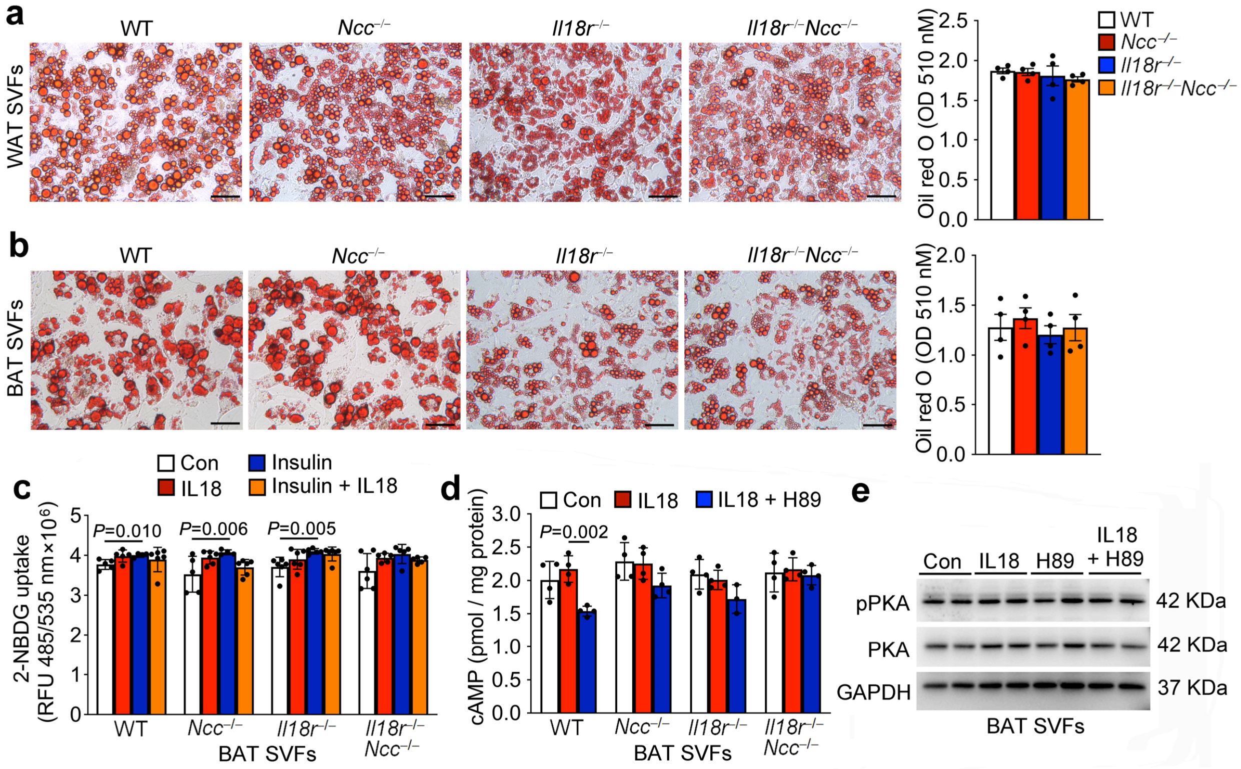

Supplementary Fig. 5. Pre-adipocyte adipogenesis and adipocyte glucose uptake and cAMP signaling from WT, *Ncc*$^{-/-}$, *Il18r*$^{-/-}$ and *Il18r*$^{-/-}$*Ncc*$^{-/-}$ mice. **a**/**b**. Oil-red O staining and quantification of lipid accumulation (OD 510 nm) in differentiated white adipocytes (**a**) and differentiated brown adipocytes (**b**), scale: 50 μm (n=4 biologically independent samples per group). **c**. Glucose (2-NBDG) uptake in differentiated brown adipocytes from different mice with or without 20 nM insulin and 0 or 100 ng/mL of IL18 treatment for 24 hrs as indicated (WT: n=6; *Ncc*$^{-/-}$: n=6, *Il18r*$^{-/-}$: n=5; *Il18r*$^{-/-}$*Ncc*$^{-/-}$: n=6 biologically independent samples). **d-e**. cAMP levels (WT: n=4; *Ncc*$^{-/-}$: n=4, *Il18r*$^{-/-}$: n=4; *Il18r*$^{-/-}$*Ncc*$^{-/-}$: n=3 biologically independent samples) (**d**) and immunoblot and quantification of pPKA to total PKA (**e**) in differentiated brown adipocytes from different mice with or without 100 ng/mL of IL18, 20 nM insulin, or 20 μM H89. Data are mean±SEM, one-way ANOVA test, followed by LSD post-test.

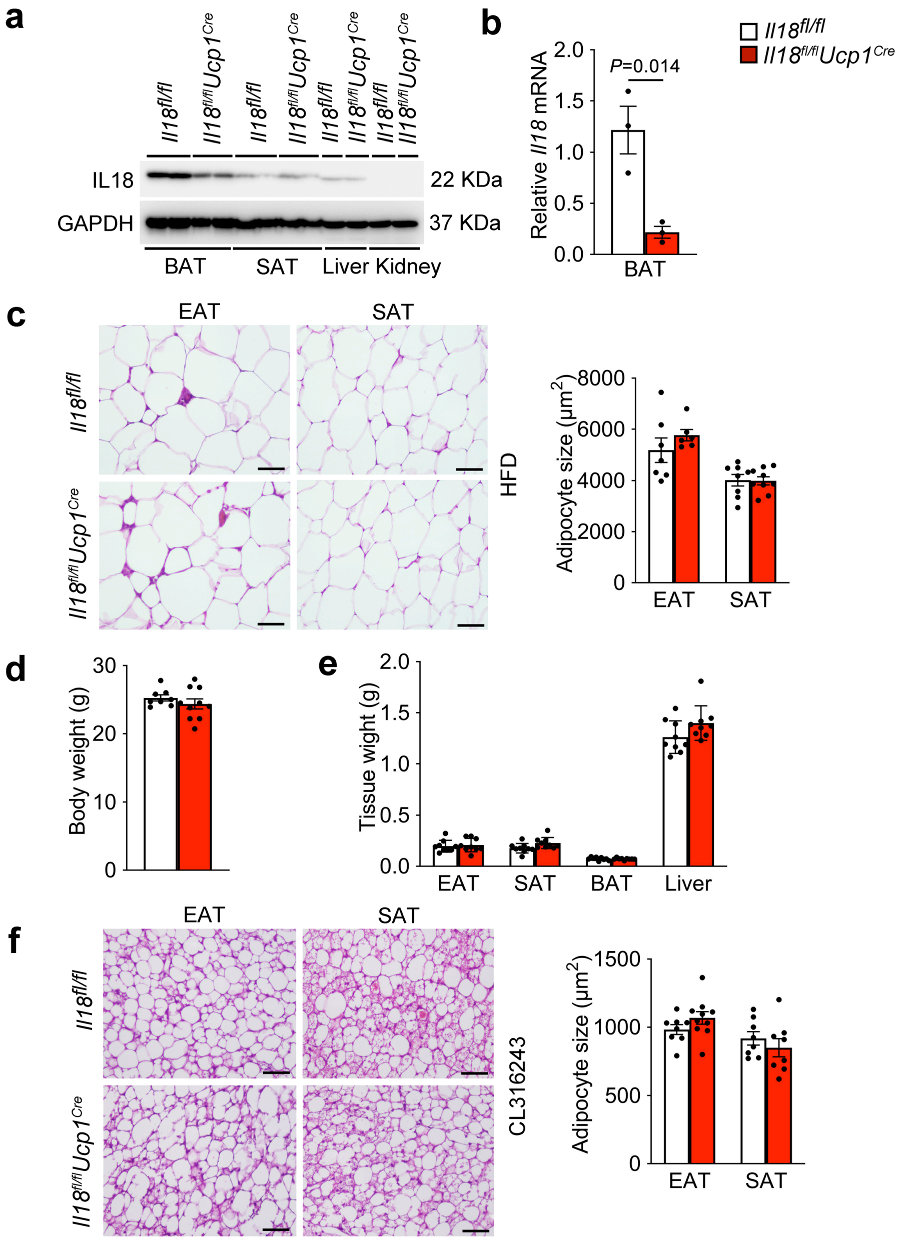

Supplementary Fig. 6. IL18 expression, adipocyte morphology, and body and tissue weights in $Il18^{fl/fl}$ and $Il18^{fl/fl}Ucp1^{Cre}$ mice. **a**. Immunoblot analysis of IL18 expression in different tissues from LFD-fed $Il18^{fl/fl}$ and $Il18^{fl/fl}Ucp1^{Cre}$ mice. Representative of 3 independent experiments. **b**. RT-PCR analysis of *Il18* in BAT from LFD-fed $Il18^{fl/fl}$ and $Il18^{fl/fl}Ucp1^{Cre}$ mice (n=3 biologically independent samples per group). **c**. Representative images of H&E staining of EAT and SAT from mice fed a HFD for 12 weeks ($Il18^{fl/fl}$-EAT: n=7; $Il18^{fl/fl}Ucp1^{Cre}$-EAT: n=6; $Il18^{fl/fl}$-EAT: n=8; $Il18^{fl/fl}Ucp1^{Cre}$-EAT: n=9 biologically independent samples). Scale: 50 μm. Bodyweight (**d**), tissue weight (**e**), and representative H&E staining of EAT and SAT (**f**) from mice after 7 days of CL316243 treatment (n=9 biologically independent samples per group). Scale: 50 μm. Data are mean±SEM, two-sided Student's t-test.

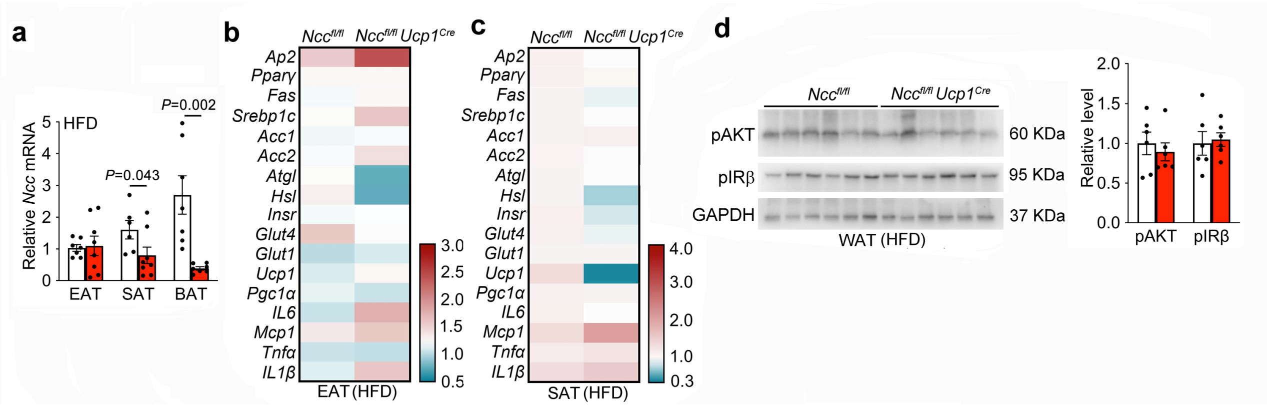

Supplementary Fig. 7. NCC and metabolic gene expression and insulin signaling in *Ncc*$^{fl/fl}$ and *Ncc*$^{fl/fl}$*Ucp1*^{Cre} mice after 12 weeks of a HFD. **a.** RT-PCR analysis of NCC expression in EAT, SAT, and BAT from *Ncc*$^{fl/fl}$ and *Ncc*$^{fl/fl}$*Ucp1*^{Cre} mice (n=6 biologically independent samples per group). **b-c**. RT-PCR analysis of lipogenic, lipolytic, glucose metabolic, thermogenic, and inflammatory genes in EAT (**b**) and SAT (**c**) from indicated mice (*Ncc*$^{fl/fl}$: n=12; *Ncc*$^{fl/fl}$*Ucp1*^{Cre}: n=9 biologically independent samples). **d**. Immunoblots analysis of pAKT, pIRβ and GAPDH in WAT from HFD-fed *Ncc*$^{fl/fl}$ and *Ncc*$^{fl/fl}$*Ucp1*^{Cre} mice (n=6 biologically independent samples per group). Data are mean±SEM, two-sided Mann-Whitney *U* test.

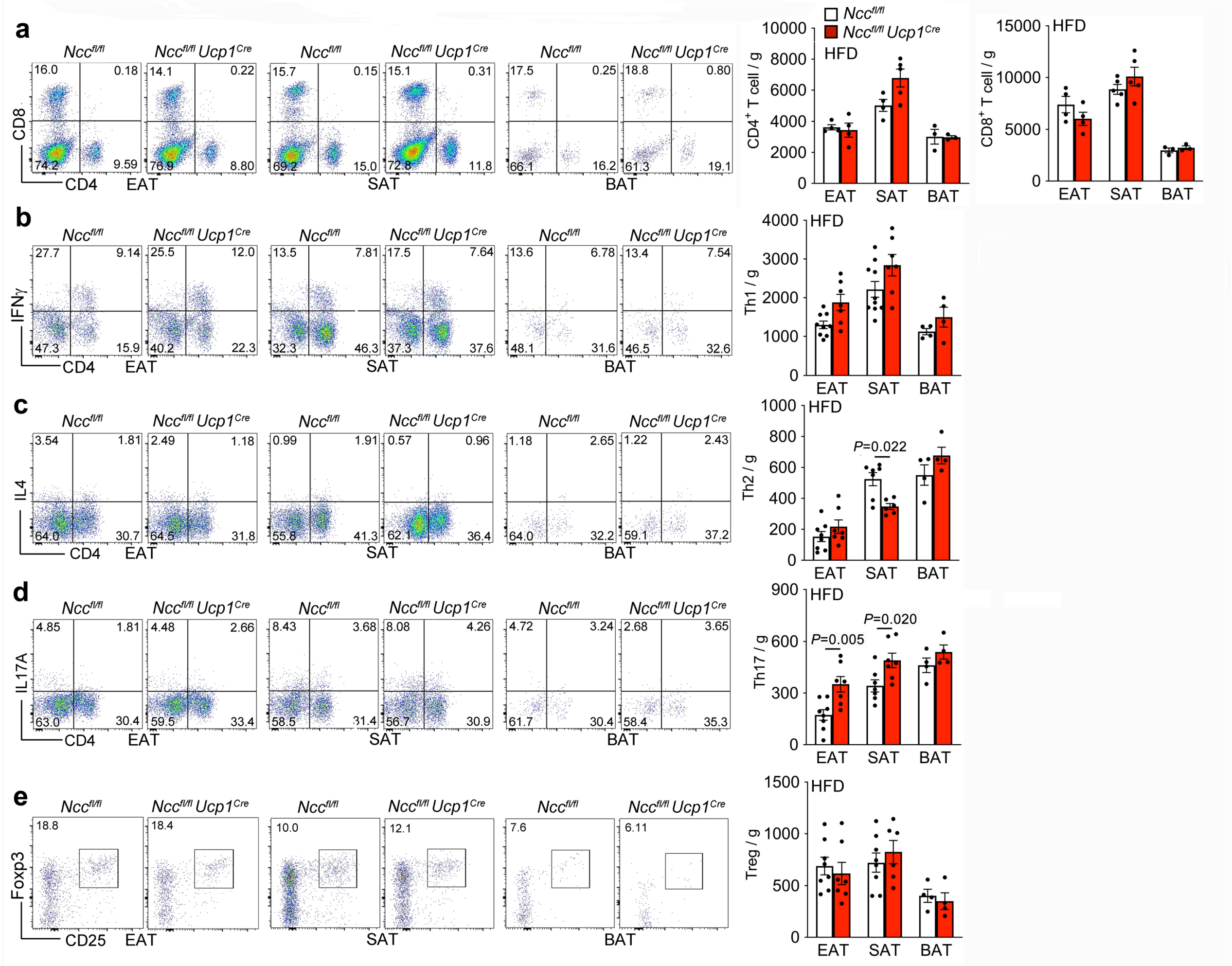

Supplementary Fig. 8. Quantification of different T cells in EAT, SAT, and BAT from *Ncc*$^{fl/fl}$ and *Ncc*$^{fl/fl}$*Ucp1*^{Cre} mice after 12 weeks of a HFD. **a-e**. Representative FACS images and quantification of total CD4$^+$ and CD8$^+$ T cells (n=4 biologically independent samples per group in EAT and SAT, n=3 biologically independent samples per group in BAT) (**a**) and CD4$^+$IFNγ$^+$ Th1, CD4$^+$IL4$^+$ Th2, CD4$^+$IL17A$^+$ Th17 (**b-d**), and CD4$^+$CD25$^+$Foxp3$^+$ Treg (n=7 biologically independent samples per group in EAT and SAT, n=4 biologically independent samples per group in BAT) (**e**) cells in EAT, SAT, and BAT from indicated mice. Data are mean±SEM, **P*<0.05, ***P*<0.01, two-sided Student's t-test.

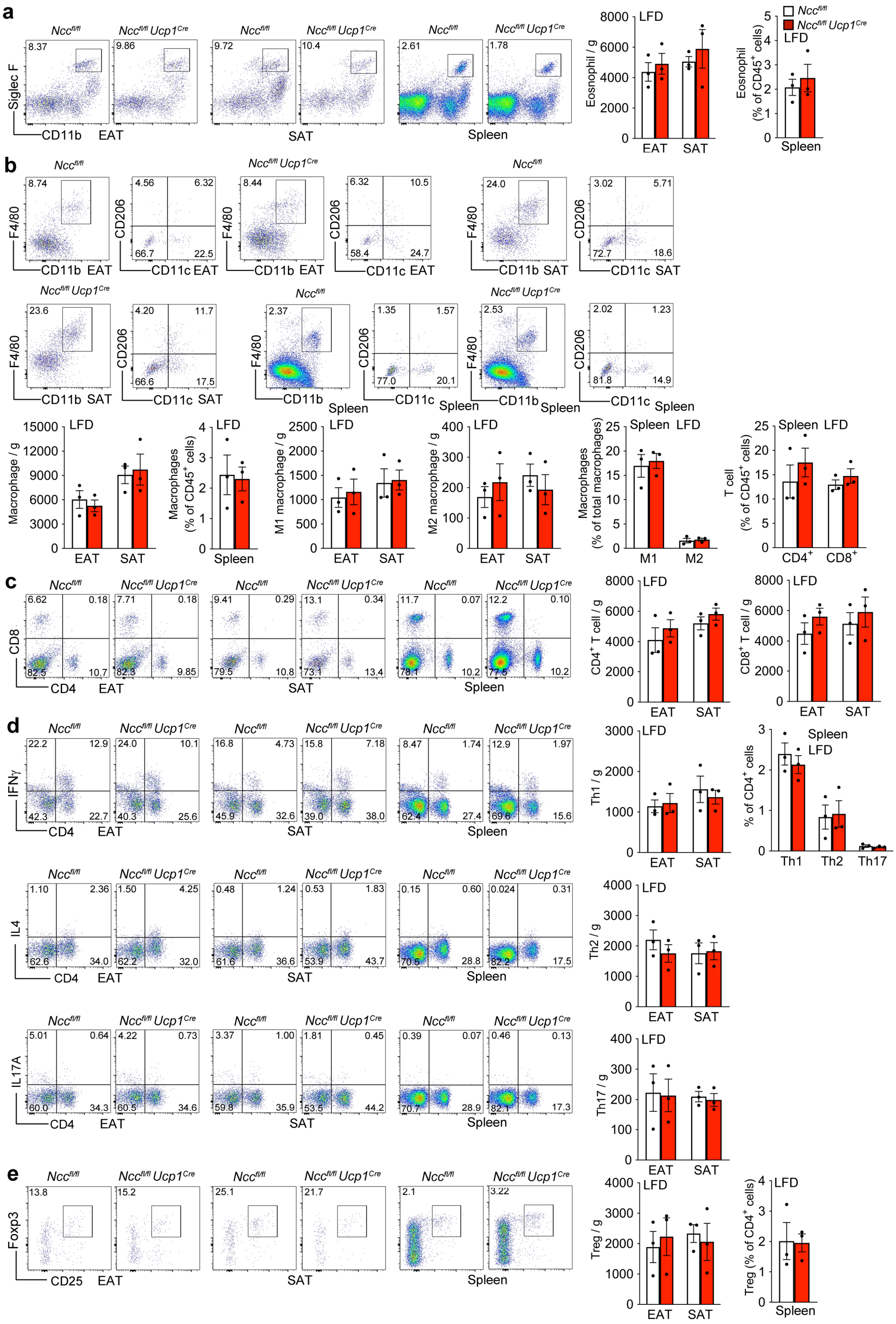
a
Nccfl/fl
Nccfl/fl Ucp1Cre
Siglec F
CD11b
EAT
SAT
Spleen
Eosnophil / g
LFD
Eosnophil (% of CD45+ cells)
b
F4/80
CD206
CD11c
Macrophage / g
Macrophages (% of CD45+ cells)
M1 macrophage / g
M2 macrophage / g
Macrophages (% of total macrophages)
M1
M2
T cell (% of CD45+ cells)
CD4+
CD8+
c
CD8
CD4
CD4+ T cell / g
CD8+ T cell / g
d
IFNγ
Th1 / g
% of CD4+ cells
Th1
Th2
Th17
IL4
Th2 / g
IL17A
Th17 / g
e
Foxp3
CD25
Treg / g
Treg (% of CD4+ cells)

Supplementary Fig. 9. Quantification of eosinophils, macrophages, and different T cells in EAT, SAT, and spleens from LFD-fed $Ncc^{fl/fl}$ and $Ncc^{fl/fl}Ucp1^{Cre}$ mice. **a-b.** Representative FACS images and quantification of $CD11b^{+}Siglec\text{-}F^{+}$ eosinophils (**a**) and $CD11b^{+}F4/80^{+}$ total macrophages, $CD11b^{+}F4/80^{+}CD11c^{+}$ M1 macrophages, and $CD11b^{+}F4/80^{+}CD206^{+}$ M2 macrophages in EAT, SAT, and BAT (**b**) from indicated mice. **c-e**, Representative FACS images and quantification of total $CD4^{+}$ and $CD8^{+}$ T cells (**c**) and $CD4^{+}IFN\gamma^{+}$ Th1, $CD4^{+}IL4^{+}$ Th2, $CD4^{+}IL17A^{+}$ Th17 T cells (**d**), and $CD4^{+}CD25^{+}Foxp3^{+}$ Treg (**e**) in EAT, SAT, and BAT from indicated mice. n=3 biologically independent samples per group. Data are mean±SEM. Two-sided Mann-Whitney *U* test.

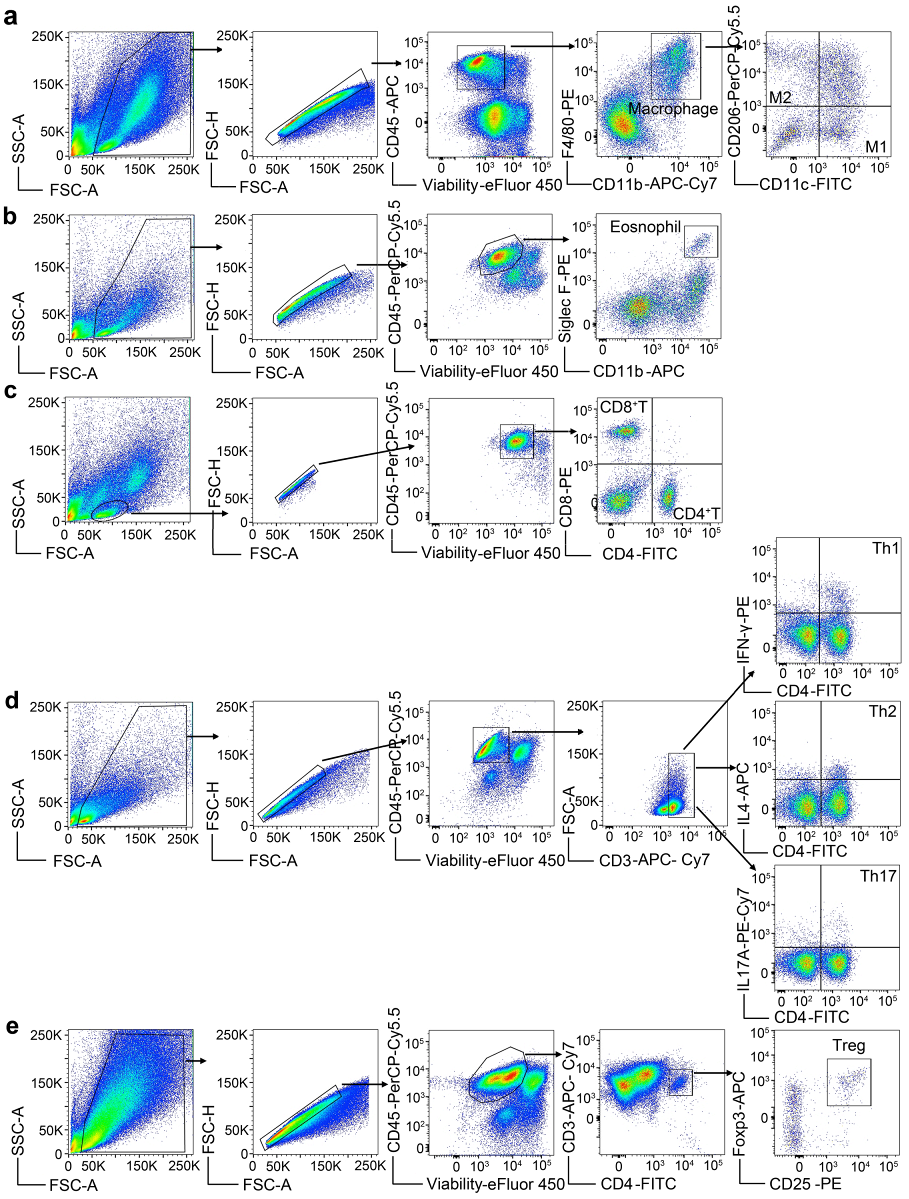

Supplementary Fig. 10. Gating strategies used for flow cytometry. **a**. Gating strategy to detect adipose tissue total ($CD45^+CD11b^+F4/80^+$), M1 ($CD45^+CD11b^+F4/80^+CD11c^+CD206^-$), and M2 ($CD45^+CD11b^+F4/80^+$ $CD11c^-CD206^+$) macrophages. **b**. Gating strategy to detect adipose tissue eosinophils ($CD45^+CD11b^+$Siglec-F^+). **c**. Gating strategy to detect adipose tissue $CD8^+$ ($CD45^+CD8^+CD4^-$) and $CD4^+$ ($CD45^+CD8^-CD4^+$) T cells. **d**. Gating strategy to detect adipose tissue Th1 ($CD45^+CD3^+CD4^+$IFN-γ^+), Th2 ($CD45^+CD3^+CD4^+IL4^+$), and Th17 ($CD45^+CD3^+CD4^+IL17A^+$) cells. **e**. Gating strategy to detct adipose tissue and splenic Treg ($CD45^+CD3^+CD4^+CD25^+Foxp3^+$) cells.

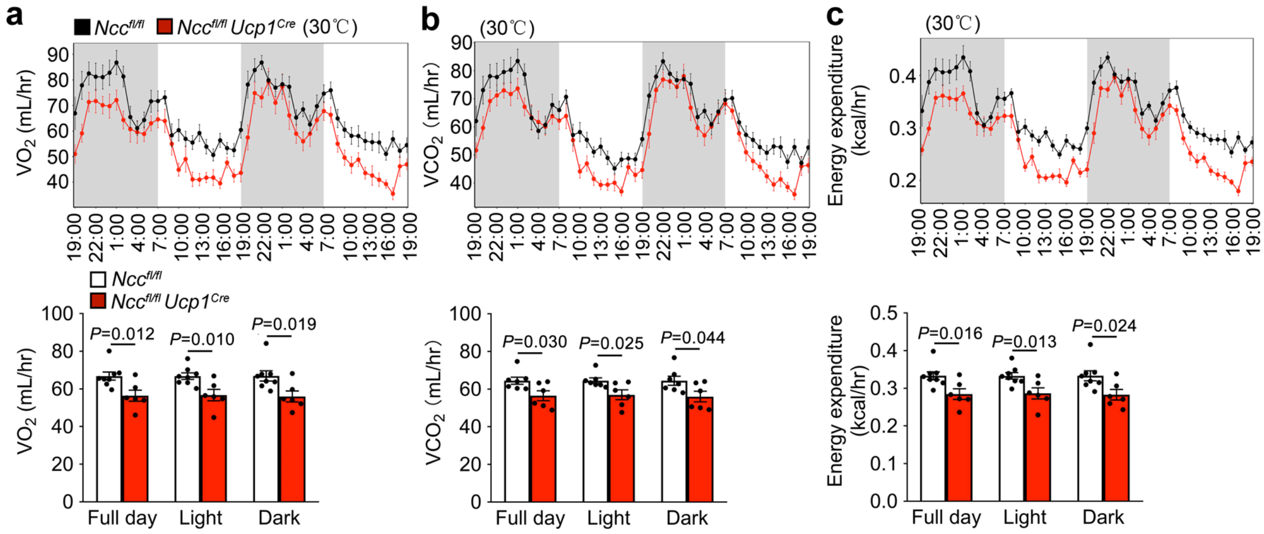

Supplementary Fig. 11. Energy expenditure in LFD-fed mice that were housed at a thermoneutral temperature (30 °C) for 7 days. **a-c.** Mouse metabolic parameters, including oxygen consumption (VO_2) (**a**) and carbon dioxide production (VCO_2) (**b**), and energy expenditure (**c**) and their average values from full day cycle, light cycle, and dark cycle during 48 hrs of monitoring in LFD-fed in *Ncc*$^{fl/fl}$ and *Ncc*$^{fl/fl}$*Ucp1*^{Cre} mice (*Ncc*$^{fl/fl}$: n=8; *Ncc*$^{fl/fl}$*Ucp1*^{Cre}: n=6 biologically independent animals). Data are mean±SEM, two-sided Student's t-test.

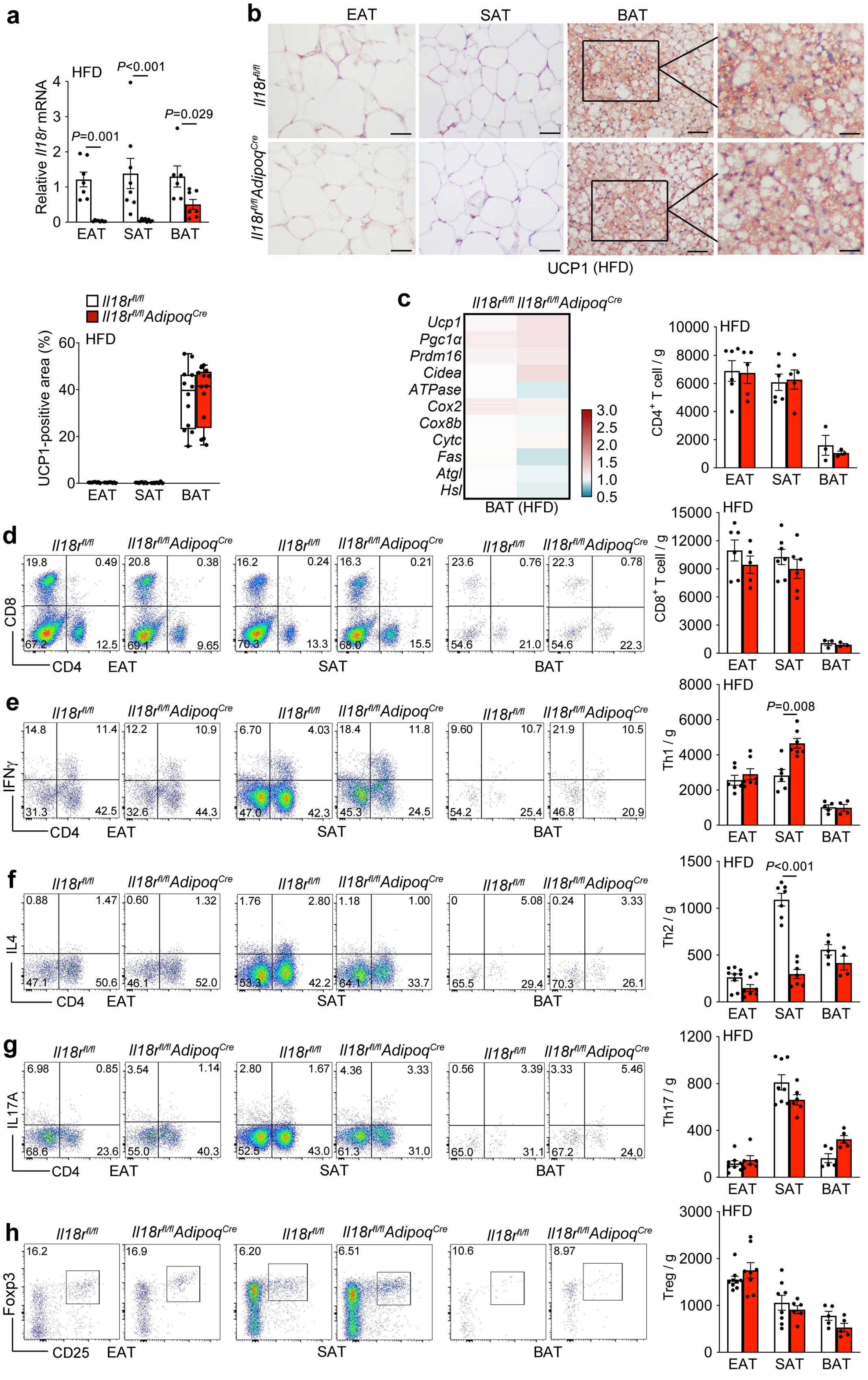
a
HFD
Relative Il18r mRNA
P=0.001
P<0.001
P=0.029
EAT
SAT
BAT
b
EAT
SAT
BAT
Il18r^fl/fl
Il18r^fl/fl AdipoqCre
UCP1 (HFD)
□ Il18r^fl/fl
■ Il18r^fl/fl AdipoqCre
HFD
UCP1-positive area (%)
EAT
SAT
BAT
c
Il18r^fl/fl Il18r^fl/fl AdipoqCre
Ucp1
Pgc1α
Prdm16
Cidea
ATPase
Cox2
Cox8b
Cytc
Fas
Atgl
Hsl
3.0
2.5
2.0
1.5
1.0
0.5
BAT (HFD)
HFD
CD4+ T cell / g
EAT
SAT
BAT
HFD
CD8+ T cell / g
EAT
SAT
BAT
d
CD8
CD4
EAT
SAT
BAT
e
IFNγ
CD4
EAT
SAT
BAT
HFD
P=0.008
Th1 / g
f
IL4
CD4
EAT
SAT
BAT
HFD
P<0.001
Th2 / g
g
IL17A
CD4
EAT
SAT
BAT
HFD
Th17 / g
h
Foxp3
CD25
EAT
SAT
BAT
HFD
Treg / g

Supplementary Fig. 12. IL18r expression, UCP1 immunostaining, thermogenic gene expression, and different T-cell numbers in $Il18r^{fl/fl}$ and $Il18r^{fl/fl}Adipoq^{Cre}$ mice after 12 weeks of a HFD. **a.** RT-PCR analysis of IL18r expression in EAT, SAT, and BAT from $Il18r^{fl/fl}$ and $Il18r^{fl/fl}Adipoq^{Cre}$ mice (n=6 biologically independent samples per group). **b.** Immunostaining and quantification of UCP1-positive area in EAT, SAT, and BAT ($Il18r^{fl/fl}$-EAT: n=8; $Il18r^{fl/fl}Adipoq^{Cre}$-EAT: n=9; $Il18r^{fl/fl}$-SAT: n=8; $Il18r^{fl/fl}Adipoq^{Cre}$-SAT: n=7; $Il18r^{fl/fl}$-BAT: n=12; $Il18r^{fl/fl}Adipoq^{Cre}$-BAT: n=13 biologically independent samples). Scale: 50 μm, inset: 25 μm. **c**. RT-PCR analyses of thermogenic genes in BAT from $Il18r^{fl/fl}$ and $Il18r^{fl/fl}Adipoq^{Cre}$ mice (n=6 biologically independent samples per group). **d-h**. Representative FACS images and quantification of total $CD4^+$ and $CD8^+$ T cells ($Il18r^{fl/fl}$-EAT: n=6; $Il18r^{fl/fl}Adipoq^{Cre}$-EAT: n=5; $Il18r^{fl/fl}$-SAT: n=6; $Il18r^{fl/fl}Adipoq^{Cre}$-SAT: n=5; $Il18r^{fl/fl}$-BAT: n=3; $Il18r^{fl/fl}Adipoq^{Cre}$-BAT: n=3 biologically independent samples) (**d**) and $CD4^+IFN\gamma^+$ Th1, $CD4^+IL4^+$ Th2, $CD4^+IL17A^+$ Th17 ($Il18r^{fl/fl}$-EAT: n=6; $Il18r^{fl/fl}Adipoq^{Cre}$-EAT: n=7; $Il18r^{fl/fl}$-SAT: n=6; $Il18r^{fl/fl}Adipoq^{Cre}$-SAT: n=7; $Il18r^{fl/fl}$-BAT: n=5; $Il18r^{fl/fl}Adipoq^{Cre}$-BAT: n=5 biologically independent samples) (**e-g**), and $CD4^+CD25^+Foxp3^+$ Treg ($Il18r^{fl/fl}$-EAT: n=8; $Il18r^{fl/fl}Adipoq^{Cre}$-EAT: n=6; $Il18r^{fl/fl}$-SAT: n=8; $Il18r^{fl/fl}Adipoq^{Cre}$-SAT: n=7; $Il18r^{fl/fl}$-BAT: n=5; $Il18r^{fl/fl}Adipoq^{Cre}$-BAT: n=5 biologically independent samples) (**h**) in EAT, SAT, and BAT from indicated mice. Data are mean±SEM, two-sided Mann-Whitney *U* test.

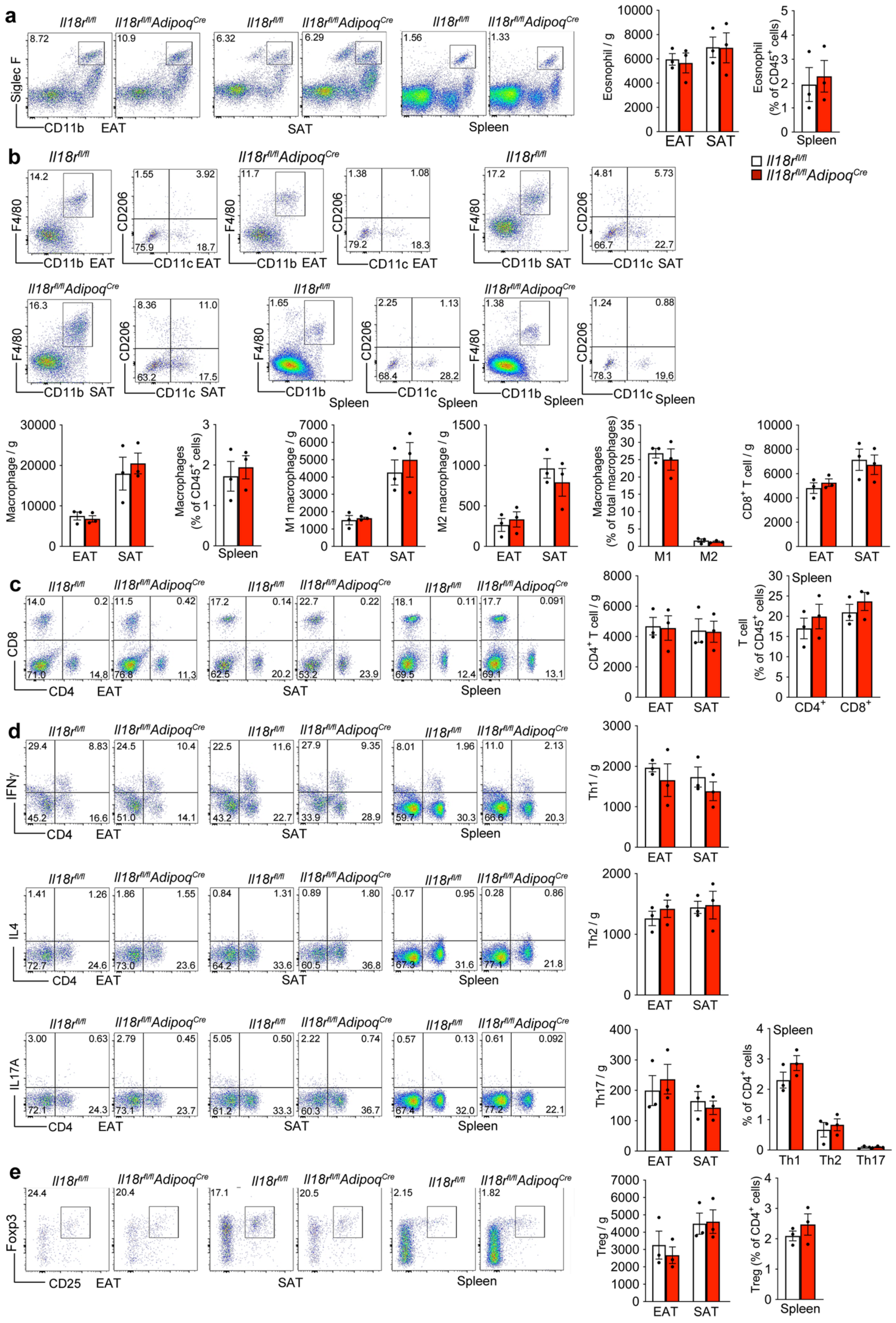
a
Il18rfl/fl
Il18rfl/flAdipoqCre
Siglec F
CD11b
EAT
SAT
Spleen
Eosnophil / g
Eosnophil (% of CD45+ cells)
b
F4/80
CD206
CD11c
M1 macrophage / g
M2 macrophage / g
Macrophage / g
Macrophages (% of CD45+ cells)
Macrophages (% of total macrophages)
CD8+ T cell / g
c
CD8
CD4
CD4+ T cell / g
T cell (% of CD45+ cells)
d
IFNγ
IL4
IL17A
Th1 / g
Th2 / g
Th17 / g
% of CD4+ cells
e
Foxp3
CD25
Treg / g
Treg (% of CD4+ cells)

Supplementary Fig. 13. FACS analyses of eosinophils, macrophages, and different T cells in EAT, SAT, and spleens from LFD-fed *Il18r*$^{fl/fl}$ and *Il18r*$^{fl/fl}$*Adipoq*^{Cre} mice. **a-b.** Representative FACS images and quantification of CD11b$^+$Siglec-F$^+$ eosinophils (**a**) and CD11b$^+$F4/80$^+$ total macrophages, CD11b$^+$F4/80$^+$CD11c$^+$ M1 macrophages, and CD11b$^+$F4/80$^+$CD206$^+$ M2 macrophages in EAT, SAT, and spleens (**b**) from indicated mice. **c-e**. Representative FACS images and quantification of total CD4$^+$ and CD8$^+$ T cells (**c**), CD4$^+$IFNγ$^+$ Th1, CD4$^+$IL4$^+$ Th2, CD4$^+$IL17A$^+$ Th17 (**d**), and CD4$^+$CD25$^+$Foxp3$^+$ Treg (**e**) cells in EAT, SAT, and spleens from indicated mice. n=3 biologically independent per group. Data are mean±SEM.

Supplementary Table 1. Antibodies dilutions and sources.

Antibodies	Dilutions, Applications	Catalog Numbers, Vendors
Rabbit anti-IL18	1:25, immunohistology	ab207324, Abcam
Goat anti-IL18r	1:20, immunohistology	AF856, R&D Systems
Rabbit anti-NCC	1:50, immunohistology	AB3553, Millipore
Mouse anti-UCP1	1:250, immunohistology	MAB6158, R&D Systems
Rat anti-Mac-3	1:200, immunohistology	108502, BioLegend
Biotin-conjugated secondary antibodies	1:500, immunohistology	Vector Laboratories
HRP-streptavidin	1:500, immunohistology	P039701-2, DAKO
Mouse anti-IRβ antibodies	1:50, immunohistology	3020S, Cell Signaling Tech
Alex Fluor 555	1:500, immunohistology	Thermo Fisher Scientific
Alex Fluor 488	1:300, immunohistology	Thermo Fisher Scientific
Rabbit anti-IL18	1:1000, immunoblot	ab207324, Abcam
Goat anti-IL18r	1:1000, immunoblot	AF856, R&D Systems
Rabbit anti-NCC	1:1000, immunoblot	AB3553, Millipore
Mouse anti-UCP1	1:1000, immunoblot	MAB6158, R&D Systems
Rabbit anti-PGC1α	1:1000, immunoblot	ab54481, Abcam
Rabbit anti-ATGL	1:1000, immunoblot	PA5-17436, Invitrogen
Rabbit anti-β3-AR	1:1000, immunoblot	PA5-117769, Thermo Fisher Scientific
Goat anti-GLUT4	1:1000, immunoblot	sc-1608, Santa Cruz Biotech
Mouse anti-PPARγ	1:1000, immunoblot	sc-7273, Santa Cruz Biotech
Rabbit anti-pAMPK	1:1000, immunoblot	4185, Cell Signaling Tech
Rabbit anti-AMPK	1:1000, immunoblot	2532, Cell Signaling Tech
Rabbit anti-pHSL	1:1000, immunoblot	4139, Cell Signaling Tech
Rabbit anti-HSL	1:1000, immunoblot	4107, Cell Signaling Tech
Rabbit anti-COX IV	1:1000, immunoblot	4850, Cell Signaling Tech
Rabbit anti-Cyt C	1:1000, immunoblot	11940, Cell Signaling Tech
Rabbit anti-pAKT	1:1000, immunoblot	4060, Cell Signaling Tech
Rabbit anti-AKT	1:1000, immunoblot	9272, Cell Signaling Tech
Rabbit anti-pIRβ	1:1000, immunoblot	3026, Cell Signaling Tech
Mouse anti-IRβ	1:1000, immunoblot	3020, Cell Signaling Tech
Rabbit anti-pPKA	1:1000, immunoblot	4781, Cell Signaling Tech
Rabbit anti-PKA	1:1000, immunoblot	4782, Cell Signaling Tech
Rabbit anti-GAPDH	1:1000, immunoblot	2118, Cell Signaling Tech
Rabbit anti-β-actin	1:1000, immunoblot	8457, Cell Signaling Tech

HRP-conjugated secondary antibodies	1:5000, immunoblot	Thermo Fisher Scientific
Viability Dye eFluor 450	1:100, flow cytometry	65-0863-14, eBioscience
Anti-CD45-APC	1:100, flow cytometry	103112, BioLegend
Anti-CD11b-APC-Cyanine7	1:100, flow cytometry	101226, BioLegend
Anti-F4/80-PE	1:100, flow cytometry	123110, BioLegend
Anti-CD206-PerCP-Cyanine5.5	1:100, flow cytometry	141716, BioLegend
Anti-CD11c-FITC	1:100, flow cytometry	53-0114-82, eBioscience
Anti-CD45-PerCP-Cyanine5.5	1:100, flow cytometry	45-0451-82, Invitrogen
Anti-CD11b-APC	1:100, flow cytometry	17-0112-83, eBioscience
Anti-Siglec-F-PE	1:100, flow cytometry	12-1702-82, eBioscience
Anti-CD4-FITC	1:100, flow cytometry	11-0042-85, eBiosciences
Anti-CD8-PE	1:100, flow cytometry	100708, BioLegend
Anti-CD3-APC- Cyanine7	1:100, flow cytometry	100222, BioLegend
Anti-CD25-PE	1:100, flow cytometry	102008, BioLegend
Anti-Foxp3-APC	1:100, flow cytometry	17-5773-82, eBioscience
Anti-IFN-γ-PE	1:100, flow cytometry	12-7311-82, eBioscience
Anti-IL4-APC	1:100, flow cytometry	17-7041-82, eBioscience
Anti-IL17A-PE-Cyanine7	1:100, flow cytometry	506922, BioLegend

Supplementary Table 2. Real-time PCR primer sequences.

Gene	Forward primers (5' to 3')	Reverse primers (5' to 3')
Ucp1	CACTCAGGATTGGCCTCTACG	GGGGTTTGATCCCATGCAGA
Prdm16	CCACCAGACTTCGAGCTACG	ACACCTCTGTATCCGTCAGCA
Pgc1α	CCCTGCCATTGTTAAGACC	TGCTGCTGTTCCTGTTTTC
Elovl3	TTCTCACGCGGGTTAAAAATGG	GAGCAACAGATAGACGACCAC
Tmem26	ACCCTGTCATCCCACAGAG	TGTTTGGTGGAGTCCTAAGGTC
CD137	CGTGCAGAACTCCTGTGATAAC	GTCCACCTATGCTGGAGAAGG
Cited1	ACTAGCTCCTCTGGATCGACA	GACCCAGTTTTGCATGGGC
Pdgfrα	AGCAGGCAGGGCTTCAACGG	ACACAGTCTGGCGTGCGTCC
Ap2	ACACCGAGATTTCCTTCA AACTG	CCATCTAGGGTTATGATGCTCTTCA
Pparγ	TTGTAGAGTGCCAGGTGCTG	CCTCCATAGCTCAGGTGGAA
Fas	AAGTCCCAGAAATCGCCTATG	GGTATGGTTTCACGACTGGAG
Srebp1c	ATGCCATGGGCAAGTACACA	ATAGCATCTCCTGCGCACTC
Acc1	TGTCCACCCAAGCATTTCTTC	CATCCAACACCAGTTCAGTATACGT
Acc2	ACTTTGACCTGACCGCTGTG	CTGAGTGCCGGATAATGGC
Atgl	GGATGAAAGAGCAGACGGGTAG	CGCAAGACAGTGGCACAGAG
Hsl	ACTGAGATTGAGGTGCTGTC	AGGTGAGATGGTAACTGTGAG
Insr	CTACAGTGTTCGAGTCCGGG	TGGCAATATTTGATGGGACATCT
Glut4	CTATTCAACCAGCATCTTCGAG	CTACTAAGAGCACCGAGACC
Glut1	GACGGGCCGCCTCATGTTGG	GCTCTCCGTAGCGGTGGTTCC
IL18	CAGGCCTGACATCTTCTGCAA	CTGACATGGCAGCCATTGT
Ncc	CTTCGGCCACTGGCATTCTG	GATGGCAAGGTAGGAGATGG
Il18r	TTAGGACCAAAGTGT-GAGAAGG	TCTCGTCTCTTT-CCGCTATGCG
IL6	CCTTCCTACCCCAATTTCCAA	AGATGAATTGGATGGTCTTGGTC
Mcp1	TTAAAAACCTGGATCGGAACCAA	GCATTAGCTTCAGATTTACGGGT
Tnfα	CAAAGGGAGAGTGGTCAGGT	GGCAACAAGGTAGAGAGGC
IL1β	TGAAATGCCACCTTTTGACAG	CCACAGCCACAATGAGTGATAC
Arg1	CTCCAAGCCAAAGTCCTTAGAG	AGGAGCTGTCATTAGGGACATC
Mrc1	TGATTACGAGCAGTGGAAGC	GTTCACCGTAAGCCCAATTT
Clec10a	CTCTGGAGAGCACAGTGGAG	ACTTCCGAGCCGTTGTTCT
Siglec-F	CTGGCTACGGACGGTTATTCG	GGAATTGGGGTACTGGACTTG
IL4	GGTCTCAACCCCCAGCTAGT	GCCGATGATCTCTCTCAAGTGAT
IL13	CCTGGCTCTTGCTTGCCTT	GGTCTTGTGTGATGTTGCTCA
ATPase	CACCACCAAGAAGGGATCGA	GCAGGGTCAGTCAGGTCATCA

Cox2	TCTCCCCTCTCTACGCATTCT	TCATTGGTGCCCTATGGTTT
Cox8b	TGCGAAGTTCACAGTGGTTC	ATGCTGCGGAGCTCTTTTTA
Cytc	GGCTGCTGGATTCTCTTACACA	CCAAATACTCCATCAGGGTATCCT
18s rRNA	TCATAAGCTTGCGTTGATTA	TAGTCAAGTTCGACCGTCTT
16s rRNA	ACATCCCAATGGTGTAGAAG	AAGTTGAGAGCGCTTATTTG
Pten	TGGATTCGACTTAGACTTGACCT	GCGGTGTCATAATGTCTCTCAG
Stat6	ACCTGTCCATTCGCTCACTG	ATCTGGGGCTCTGGAGTAGG
Pdpn	ACCGTGCCAGTGTTGTTCTG	AGCACCTGTGGTTGTTATTTTGT
Zbtb16	CTGGGACTTTGTGCGATGTG	CGGTGGAAGAGGATCTCAAACA
GAPDH	TGTCATACTTGGCAGGTTTCT	CGTGTTCCTACCCCCAATGT

Supplementary Table 3. Human donor information for Fig. 5o.

Donor number	Procedure	Age	Gender	BMI	Diabetic	Tissue	Source
1	Panniculectomy	49	Male	32.5	No	Abdomen	Brigham and Women's Hospital
2	Abdominoplasty	50	Female	21.3	No	Abdomen	Brigham and Women's Hospital
