# Supplementary material for: Differential IL18 signaling via IL18 receptor and Na-Cl co-transporter discriminating thermogenesis and glucose metabolism regulation
Source: Nat Commun. 2022 Dec 8;13:7582. doi: 10.1038/s41467-022-35256-8 (PMC9732325; doi:10.1038/s41467-022-35256-8)
Supplement: Supplementary file 2 — Reporting Summary [file 41467_2022_35256_MOESM2_ESM.pdf]

nature portfolio

Corresponding author(s): Guo-Ping Shi; Zhangsuo Liu

Last updated by author(s): Nov 12, 2022

Reporting Summary

Nature Portfolio wishes to improve the reproducibility of the work that we publish. This form provides structure for consistency and transparency in reporting. For further information on Nature Portfolio policies, see our Editorial Policies and the Editorial Policy Checklist.

Statistics

For all statistical analyses, confirm that the following items are present in the figure legend, table legend, main text, or Methods section.

n/a	Confirmed	
☐	☒	The exact sample size (n) for each experimental group/condition, given as a discrete number and unit of measurement
☐	☒	A statement on whether measurements were taken from distinct samples or whether the same sample was measured repeatedly
☐	☒	The statistical test(s) used AND whether they are one- or two-sided<br>*Only common tests should be described solely by name; describe more complex techniques in the Methods section.*
☐	☒	A description of all covariates tested
☐	☒	A description of any assumptions or corrections, such as tests of normality and adjustment for multiple comparisons
☐	☒	A full description of the statistical parameters including central tendency (e.g. means) or other basic estimates (e.g. regression coefficient) AND variation (e.g. standard deviation) or associated estimates of uncertainty (e.g. confidence intervals)
☐	☒	For null hypothesis testing, the test statistic (e.g. F, t, r) with confidence intervals, effect sizes, degrees of freedom and P value noted<br>*Give P values as exact values whenever suitable.*
☒	☐	For Bayesian analysis, information on the choice of priors and Markov chain Monte Carlo settings
☐	☒	For hierarchical and complex designs, identification of the appropriate level for tests and full reporting of outcomes
☒	☐	Estimates of effect sizes (e.g. Cohen's d, Pearson's r), indicating how they were calculated

Our web collection on statistics for biologists contains articles on many of the points above.

Software and code

Policy information about availability of computer code

Data collection	For indirect calorimetry experiments, we used Columbus Instruments Oxymax Comprehensive Lab Animal Monitoring System (CLAMS) to obtain the raw data. Oxygen consumption rate (OCR) was performed on the Agilent Seahorse XF96 cellular Flux Analyzer.
Data analysis	CalR Version 1.3: A Web-based Analysis Tool for Indirect Calorimetry Experiments, Image-Pro Plus 6.0, FlowJo V10 and GraphPad Prism 9 were used in this study.

For manuscripts utilizing custom algorithms or software that are central to the research but not yet described in published literature, software must be made available to editors and reviewers. We strongly encourage code deposition in a community repository (e.g. GitHub). See the Nature Portfolio guidelines for submitting code & software for further information.

Data

Policy information about availability of data

All manuscripts must include a data availability statement. This statement should provide the following information, where applicable:

- Accession codes, unique identifiers, or web links for publicly available datasets
- A description of any restrictions on data availability
- For clinical datasets or third party data, please ensure that the statement adheres to our policy

Data supporting the findings of this manuscript are available from the corresponding authors upon reasonable request. The source data underlying all Figures and Supplementary Figures are provided as a Source Data file.

Field-specific reporting

Please select the one below that is the best fit for your research. If you are not sure, read the appropriate sections before making your selection.

☒ Life sciences ☐ Behavioural & social sciences ☐ Ecological, evolutionary & environmental sciences

For a reference copy of the document with all sections, see nature.com/documents/nr-reporting-summary-flat.pdf

Life sciences study design

All studies must disclose on these points even when the disclosure is negative.

Sample size	Sample sizes were assessed using the power calculation at $p<0.05$, power >0.80.
Data exclusions	No data were excluded from the analyses.
Replication	Each independent experiment was replicated at least 3-4 times.
Randomization	All mice and tissue samples grouped blindly based on mice/sample availability and collected into experimental groups randomly.
Blinding	The investigators were blinded to group allocation during data collection and analysis.

Reporting for specific materials, systems and methods

We require information from authors about some types of materials, experimental systems and methods used in many studies. Here, indicate whether each material, system or method listed is relevant to your study. If you are not sure if a list item applies to your research, read the appropriate section before selecting a response.

Materials & experimental systems

n/a	Involved in the study
☐	☒ Antibodies
☒	☐ Eukaryotic cell lines
☒	☐ Palaeontology and archaeology
☐	☒ Animals and other organisms
☐	☒ Human research participants
☒	☐ Clinical data
☒	☐ Dual use research of concern

Methods

n/a	Involved in the study
☒	☐ ChIP-seq
☐	☒ Flow cytometry
☒	☐ MRI-based neuroimaging

Antibodies

Antibodies used	Rabbit anti-IL18 (ab207324; clone name: EPR19954) and rabbit anti-PGC1α (ab54481) are from Abcam, Cambridge, MA.<br>Goat anti-IL18r (AF856) and mouse anti-UCP1 (MAB6158; clone name: 536435) are from R&D Systems, Minneapolis, MN.<br>Rabbit anti-NCC (AB3553) is from Millipore, Temecula, CA.<br>Rabbit anti-β3-AR (PA5-117769) and rabbit anti-ATGL (PA5-17436) is from Thermo Fisher Scientific, Tracy, CA<br>Mouse anti-IRβ antibodies (3020; clone name: L55B10), rabbit anti-pHSL (4139), rabbit anti-HSL (4107), rabbit anti-COX IV (4850; clone name: 3E11), rabbit anti-Cyt C (11940; clone name: D18C7), rabbit anti-pAKT (4060; clone name: D9E), rabbit anti-AKT (9272), rabbit anti-pIRβ (3026; clone name: 14A4), mouse anti-IRβ (3020; clone name: L55B10), rabbit anti-pAMPK (4185), rabbit anti-AMPK (2532) rabbit anti-pPKA (4781), rabbit anti-PKA (4782), rabbit anti-β-actin (8457; clone name: D6A8) and rabbit anti-GAPDH (2118; clone name: 9F3) are from Cell Signaling Technology, Danvers, MA.<br>Goat anti-GLUT4 (sc-1608), mouse anti-PPARγ (sc-7273; clone name: E-8) are from Santa Cruz Biotechnology, Inc, Dallas, TX.<br>Fixable Viability Dye eFluor 450 (65-0863-14), anti-CD11c-FITC (53-0114-82; clone name: N418), anti-CD45-PerCP-Cyanine5.5 (45-0451-82; clone name: 30-F11), anti-CD11b-APC (17-0112-83; clone name: M1/70), and anti-Siglec F-PE (12-1702-82; clone name: 1RNM44N), anti-CD4-FITC (11-0042-85; clone name: RM4-5), anti-Foxp3-APC (17-5773-82; clone name: FJK-16s), anti-IFN-γ-PE (12-7311-82; clone name: XMG1.2), anti-IL4-APC (17-7041-82; clone name: 11B11), and rabbit anti-ATGL (1:1000, PA5-17436) are from eBioscience San Diego, CA<br>Rat anti-Mac-3 (108502; clone name: M3/84), anti-CD25-PE (102008; clone name: PC61), anti-CD45-APC (103112; clone name: 30-F11), anti-CD11b-APC-Cyanine7 (101226; clone name: M1/70) and anti-F4/80-PE (123110; clone name: BM8), anti-CD206-PerCP-Cyanine5.5 (141716; clone name: C068C2), anti-CD8-PE (100708; clone name: 53-6.7), anti-CD3-APC- Cyanine7 (100222; clone name: 17A2), and anti-IL17A-PE-Cyanine7 (506922; clone name: TC11-18H10.1) are from BioLegend, San Diego, CA.
Validation	Each primary antibodies was validated using proper tissues or cells before the experiments. IL18 primary antibody was validated using wild type with Il18-/- colon and pancreas in mice by immunohistochemical analysis. IL18r and NCC primary antibodies were validated using wild type with Il18r-/- or Ncc-/- pancreas in mice by immunohistochemical analysis respectively (Dev Cell, 2022;57(12):1496-511 e6). The other primary antibodies were validated using WT adipose tissue.

Animals and other organisms

Policy information about studies involving animals; ARRIVE guidelines recommended for reporting animal research

Laboratory animals	Il18r-/- (004131, C57BL/6.129) were purchased from Jackson Laboratory, Bar Harbor, ME. Ncc-/- mice (C57BL/6) was provided by University of Cincinnati. WT, Il18r-/-, Ncc-/- were crossbred.<br>Nccfl/fl (C57BL/6) was produced from Cyagen Biosciences Inc, Santa Clara, CA. Il18fl/fl mice, and Il18rfl/fl were provided by Yale University. Nccfl/fl and l18fl/fl mice were crossed with Ucp1Cre mice (024670, C57BL/6, Jackson lab), Il18rfl/fl mice was crossed with AdipoqCre mice (028020, C57BL/6, Jackson lab).<br>We also cultured primary brown and white pre-adipocytes from C57BL/6 wild type mice,Il8r-/-, Ncc-/-, and Il8r-/-Ncc-/- mice at 12-week-old.<br>Six-week-old male mice of all strains were used to consume a HFD for 12 weeks to develop obesity and insulin resistance.
Wild animals	No wild animals were used in the study.
Field-collected samples	No field collected samples were used in the study.
Ethics oversight	All animal procedures conformed to the guide for the Care and Use of Laboratory Animal published by the US National Instituted of Health and was approved by the Brigham and Women's Hospital Standing Committee on Animals (protocol #2016N000442)

Note that full information on the approval of the study protocol must also be provided in the manuscript.

Human research participants

Policy information about studies involving human research participants

Population characteristics	N/A
Recruitment	N/A
Ethics oversight	N/A

Note that full information on the approval of the study protocol must also be provided in the manuscript.

Flow Cytometry

Plots

Confirm that:

☒ The axis labels state the marker and fluorochrome used (e.g. CD4-FITC).

☒ The axis scales are clearly visible. Include numbers along axes only for bottom left plot of group (a 'group' is an analysis of identical markers).

☒ All plots are contour plots with outliers or pseudocolor plots.

☒ A numerical value for number of cells or percentage (with statistics) is provided.

Methodology

Sample preparation	Primary stromal vascular fractions (SVFs) single cell preparation. Adipose tissues were dissected, minced and digested with collagenase D for 25 min at 37 °C. The digested tissues were filtered through a 100-µm mesh filter and centrifuged at 200 g for 10 min at 4 °C. The cell pellets were suspended with red blood cell lysis buffer for 5 min. Cells were washed with PBS and centrifuged at 1500 rpm for 5 min. FACS were performed to measure eosinophils, total, M1 and M2 macrophages, CD4+ and CD8+ T cells, Th1, Th2, Th17, and Treg cells in different adipose tissues.<br>Splenocyte isolation. The spleen was removed from the mouse, placed in a cold PBS, and grinded in 5 ml PBS before filtering through a 70-µm cell strainer. Splenocytes were collected after depleting the red blood cells using the red blood cell lysis buffer. FACS were performed to measure eosinophils, total, M1 and M2 macrophages, CD4+ and CD8+ T cells, Th1, Th2, Th17, and Treg cells in splenocytes.
Instrument	We used BD FACSCanto-AR for data collection.
Software	We used Flowjo to analyze the data.
Cell population abundance	No sorting was used in this manuscript.
Gating strategy	First, immune cells were gated on the SSC/FSC plots, then single cells were gated on the FSH/FSC plots. Interested cells were stained with different marker. Gating was determined by blank and single color-staining.

☒ Tick this box to confirm that a figure exemplifying the gating strategy is provided in the Supplementary Information.
